# Decreased diarrheal and respiratory disease in HIV exposed uninfected children following vaccination with rotavirus and pneumococcal conjugate vaccines

**DOI:** 10.1371/journal.pone.0244100

**Published:** 2020-12-21

**Authors:** Gbolahan Ajibola, Kara Bennett, Kathleen M. Powis, Michael D. Hughes, Jean Leidner, Samuel Kgole, Oganne Batlang, Mompati Mmalane, Joseph Makhema, Shahin Lockman, Roger Shapiro

**Affiliations:** 1 Botswana Harvard AIDS Institute Partnership, Gaborone, Botswana; 2 Bennett Statistical Consulting, Inc, Ballston Lake, NY, United States of America; 3 Departments of Internal Medicine and Pediatrics, Massachusetts General Hospital, Boston, Massachusetts, United States of America; 4 Department of Immunology and Infectious Diseases, Harvard T. H. Chan School of Public Health, Boston, Massachusetts, United States of America; 5 Department of Biostatistics, Harvard T. H. Chan School of Public Health, Boston, Massachusetts, United States of America; 6 Goodtables Data Consulting, LLC, Norman, Oklahoma, United States of America; 7 Division of Infectious Disease, Brigham and Women's Hospital, Boston, Massachusetts, United States of America; University of Nevada Reno School of Medicine, UNITED STATES

## Abstract

**Background:**

Rotavirus vaccine (RV) and pneumococcal vaccine (PCV) decrease diarrheal and respiratory disease incidence and severity, but there are few data about the effects of these vaccines among HIV-exposed uninfected (HEU) children.

**Methods:**

We recorded RV and PCV vaccination history in a placebo-controlled trial that studied the need for cotrimoxazole among HEU infants in Botswana (the Mpepu Study). We categorized infants by enrollment before or after the simultaneous April 2012 introduction of RV and PCV, and compared diagnoses of diarrhea and pneumonia (grade 3/4), hospitalizations, and deaths from both disease conditions through the 12-month study visit by vaccine era/status across two sites (a city and a village) by Kaplan-Meier estimates.

**Results:**

Two thousand six hundred and thirty-five HEU infants were included in this secondary analysis, of these 1689 (64%) were enrolled in Gaborone (344 pre-vaccine, 1345 vaccine) and 946 (36%) in Molepolole (209 pre-vaccine, 737 vaccine). We observed substantial reduction in hazard of hospitalization or death for reason of diarrhea and pneumonia in the vaccine era versus the pre-vaccine era in Molepolole (hazard ratio, HR = 0.44, 95% confidence interval, CI = 0.28, 0.71) with smaller reduction in Gaborone (HR = 0.91, 95% CI = 0.57, 1.45). Similar downward trends were observed for diagnoses of diarrhea and pneumonia separately during the vaccine versus pre-vaccine era.

**Conclusions:**

Although temporal confounding cannot be excluded, significant declines in the burden of diarrheal and respiratory illness were observed among HEU children in Botswana following the introduction of RV and PCV. RV and PCV may maximally benefit HEU children in rural areas with higher disease burden.

## Introduction

Vaccine preventable diseases such as diarrhea and pneumonia remain leading causes of death in children under five years of age in resource-limited settings [1–3]. Infections with *rotavirus* [4, 5] and *Streptococcus pneumoniae* [4, 6] are responsible for most severe cases of diarrhea and pneumonia in children in low income regions such as Sub-Saharan Africa which have high under-five mortality rates [7]. The persistence of vaccine-preventable illnesses [8, 9], and a high proportion of a more vulnerable population of HIV-exposed uninfected (HEU) children, are among factors known to contribute to the increased under 5 mortality in Sub-Saharan Africa [10, 11].

Rotavirus vaccine (RV) and pneumococcal conjugate vaccine (PCV) have been shown to effectively decrease diarrheal and respiratory disease incidence and severity in under-fives [4, 12], leading to their broad acceptance and use globally as childhood vaccines (including in Sub-Saharan Africa). In vaccine trials, RV has been shown to reduce cases of rotavirus associated diarrhea in vaccinated children by as much as 80–95% in high income countries (HICs) and 70–74% in low-middle income countries [13–15] while incidence of *pneumococcal pneumonia* declined by as much as 58% - 75% in children aged 2–23 months [16]. Among HEU children, studies have demonstrated good immune response following vaccination but few data exist on how this translates to measured clinical outcomes [17]. HEU children have an estimated mortality that is 2-4-fold higher than HIV-unexposed children [9], and understanding the nature of this vulnerability, and the potential for mitigation from available vaccines, is of high priority [10].

The Botswana government introduced RV and PCV (given at 2, 3 and 4 months of age) in April 2012, with guidelines promoting administration of these vaccines to all children under five through the national childhood immunization program free of charge. We assessed the impact of the introduction of RV and PCV vaccination among HEU children participating in a clinical trial in Botswana.

## Methods

### Design, procedure and data collection

We abstracted RV and PCV vaccination history from the health records of HEU infants enrolled from 2011–2015 in a double blind, randomized placebo-controlled trial (the Mpepu Study NCT01229761) in Botswana [18]. The Mpepu Study enrolled women living with HIV (WLHIV) and their HEU infants from week 26 of pregnancy through 34 days postpartum and evaluated the effect of prophylactic cotrimoxazole (CTX) on mortality among HEU children. Pregnant WLHIV were identified at public antenatal clinics or maternity wards in Gaborone (city), Molepolole (village) and Lobatse (town) in Botswana. Enrolled infants were randomized in double-blinded fashion to receive CTX (100/20mg once daily until 6 months, 200/40mg once daily thereafter) or placebo syrup starting between age 14–34 days and continued through 15 months. Infant evaluations in the study occurred at birth, randomization (age 14–34 days), age 2 and 3 months, and then every 3 months through age 18 months. At each evaluation, study staff ascertained the child’s health status through caregiver interview, physical assessment of the child, and review of the child’s medical records from which vaccination records were abstracted. For this sub-analysis, we used the Mpepu primary analysis datasets created and assessed in June 2017. Children were eligible for inclusion into this sub-analysis if they were HIV exposed but uninfected were enrolled in Gaborone or Molepolole, were randomized to the Mpepu study treatment arm and had post-randomization follow up data for specific measured outcomes. All HIV positive children, those enrolled in Lobatse as well as those lost to follow up or died prior to randomization were excluded from this analysis".

We evaluated infant outcomes by defined study era (before and after the Botswana government’s simultaneous April 2012 introduction of RV and PCV) and by actual vaccine receipt status. Infants born prior to April 2012 were deemed to be in the pre-vaccine era while those born after the first of April 2012 were deemed to be in the vaccine era. Infants were deemed to have been vaccinated with RV and PCV if their health record indicated receipt of at least 1 dose of RV or PCV. Using the Division of AIDS (DAIDS) Table for Grading and Severity of Adult and Pediatric Adverse Events 2004 [19], documented cases of diarrheal disease were graded. Infants with a diagnosis of presumed bacterial pneumonia per their medical record were deemed to have grade 3/4 (severe) pneumonia if they had documented chest x-ray findings suggestive of pneumonia or were hospitalized for treatment at the time of diagnosis. All outcomes were as determined and reported by independent hospital physicians and not by study staff.

We compared the cumulative incidence of grade 3 or 4 diarrhea and pneumonia diagnoses, and the composite of hospitalization/death due to diarrhea or pneumonia at two of the three study sites [Gaborone (city) and Molepolole (village)] from start of CTX/placebo (at age 14–34 days, defined as “baseline”) through the 12-month study visit (±45 days), by vaccine era. The third study site (Lobatse) closed in 2012 and was excluded because of low enrollment, particularly in the vaccine era.

For clarity and to include all available data, and to provide a comparison of outcomes in an “intent-to-treat” manner for the change in government vaccine policy, results are mainly presented by vaccine era. For this intent-to-treat analysis, all children in the pre-vaccine era were deemed not to have been vaccinated with either vaccine while all those in the vaccine era were assumed to have received at least one dose of either vaccine. Result based on actual vaccine status in both era; “as-treated analysis” are presented as supporting/supplementary documents.

### Ethical considerations

The Office of Human Research Administration at the T.H Chan Harvard School of Public Health and the Health Research Development Committee of the Botswana Ministry of Health and Wellness provided IRB review and approval for the Mpepu study. All enrolled women provided written informed consent prior to study participation.

### Statistical analysis

The endpoints of interest were cumulative incidence of combined outcomes of hospitalization/death due to diarrhea or pneumonia and first diarrhea/pneumonia diagnosis as well as individual outcomes of diagnosis of diarrhea and pneumonia. Analyses were performed including all children in each vaccine era with events measured between the baseline (14–34 days of life) and the 12 months study visit for the intent-to-treat analysis, while infants were included based on their actual vaccine receipt status for the as-treated analysis which considered events occurring from 4–12 months.

Kaplan-Meier survival estimates were used to compare cumulative proportions of events at 12 months of age in the two study eras and Greenwood’s formula used to calculate the standard error (SE) of the difference. For those who did not experience these events, follow-up time was calculated from baseline to the date the child was last seen in the clinic or exactly 12 months from the date of birth if the last study visit occurred beyond this age. These analyses were performed using interval-censored survival methods as specific start dates for diarrhea and pneumonia events were not always available.

The Cox proportional hazards model was used to adjust for risk factors that were associated with hospitalization and/or death for reason of diarrhea or pneumonia. The risk factors considered were study site, gestational age at delivery, birth weight, infant feeding method at birth, randomized study treatment arm, maternal antiretroviral treatment (ART) status and maternal CD4 cell count. Risk factors were first considered in univariable models, and an intermediary model was selected applying backward selection methods to these risk factors with an inclusion p-value cutoff of 0.05. Finally, two-way interactions were considered for inclusion among the selected risk factors.

## Results

### Baseline characteristics

**Fig 1:**
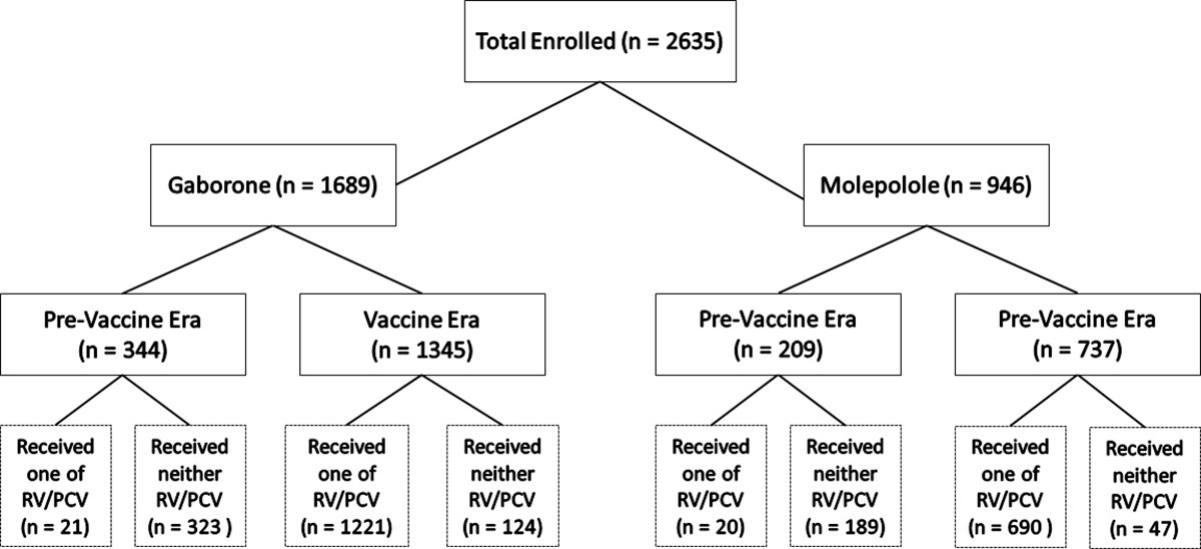
Flow diagram of study participants and vaccine receipt.

A total of 2635 HEU children were included in this analysis, 1689 (64%) in Gaborone, 946 (36%) in Molepolole. Of those in Gaborone, 1345 (80%) were born in the vaccine era and 344 (20%) in the pre-vaccine era. Similar proportions were observed in Molepolole with 737 (78%) born in the vaccine era and 209 (22%) in the pre-vaccine era. A total of 1811 (69%) infants enrolled in the study received at least 1 dose of both vaccines, 683 (26%) did not receive either of RV or PCV vaccine, 38 (1%) did not receive PCV vaccine and 103 (4%) did not receive RV vaccine (Fig 1). For clarity and to include all available data, and to provide a comparison of outcomes in an “intent-to-treat” manner for the change in government vaccine policy, results are mainly presented by vaccine era.

Birth weight and feeding method from birth did not differ between study sites. Median age at baseline was 30 days (quartiles: 29,31) in the pre-vaccine era compared with 19 days (quartiles 15,29) in the vaccine era across both site (Table 1), corresponding to modified eligibility criteria in the vaccine era implemented October 2012 that allowed randomization of younger children (from day 14–34 of life versus 28–34 before October 2012). Mothers delivering in the vaccine era had slightly higher CD4 cell counts (median 514 vaccine vs 489 pre-vaccine) and were more likely to be virally suppressed (HIV-RNA <40 copies/mL) at the time of delivery across both sites (71% vaccine era, 42% pre-vaccine).

**Table 1:** Birth and baseline demographics (N = 2,635).

Infant Characteristics		Overall (n = 2,635)		Gaborone (n = 1,689)		Molepolole (n = 946)	
		Pre-Vaccine Era	Vaccine Era	Pre-Vaccine Era	Vaccine Era	Pre-Vaccine Era	Vaccine Era
		n = 553	n = 2082	n = 344	n = 1345	n = 209	n = 737
Birth weight (kg); Median (Q1, Q3) (n = 2,635)		2.9 (2.6, 3.2)	2.9 (2.6, 3.3)	2.9 (2.6, 3.2)	2.9 (2.6, 3.3)	2.8 (2.5, 3.2)	2.9 (2.6, 3.2)
Feeding at birth (n = 2,635)	Breastfeeding only	100 (18%)	441 (21%)	47 (14%)	294 (22%)	53 (25%)	147 (20%)
	Formula feeding only	450 (81%)	1,635 (79%)	295 (86%)	1,045 (78%)	155 (74%)	590 (80%)
	Both breastfeeding and formula feeding	3 (1%)	5 (<1%)	2 (1%)	5 (<1%)	1 (<1%)	0 (0%)
	Medical complications: Infant did not feed	0 (0%)	1 (<1%)	0 (0%)	1 (<1%)	0 (0%)	0 (0%)
Days of life at baseline; Median (Q1, Q3) (n = 2,635)		30 (29, 31)	19 (15, 29)	30 (29, 31)	19 (15, 29)	30 (29, 32)	19 (15, 29)
Weight for age Z-scores at randomization; Median (Q1, Q3) (n = 2,634)		-0.9 (-1.6, - 0.1)	-0.8 (-1.5, -0.1)	-0.8 (-1.5, -0.1)	-0.75 (-1.6, -0.1)	-1.0 (-1.7, -0.2)	-0.8 (-1.4, -0.1)
**Maternal Characteristics**							
Age (years); Median (Q1, Q3) (n = 2,635)		30 (26, 34)	31 (26, 35)	29 (25, 34)	31 (26, 35)	31 (27, 34)	31 (26, 35)
CD4 count (cells/mm^3^); Median (Q1, Q3) (n = 2,598)		489 (354, 670)	514 (366, 684)	486 (346, 662)	502 (358, 675)	491 (359, 680)	532 (381, 695)
HIV RNA at delivery (copies/mL) (n = 1,303)	< = 40	230 (42%)	535 (71%)	130 (38%)	334 (68%)	100 (48%)	201 (76%)
	>40	321 (58%)	217 (29%)	212 (62%)	154 (32%)	109 (52%)	63 (24%)
ART at delivery (n = 2,635)	No documented ART in pregnancy	42 (8%)	89 (4%)	23 (7%)	65 (5%)	19 (9%)	24 (3%)
	Not on ART prior to delivery	171 (31%)	70 (3%)	134 (39%)	57 (4%)	37 (18%)	13 (2%)
	On ART prior to delivery	340 (61%)	1,923 (92%)	187 (54%)	1,223 (91%)	153 (73%)	700 (95%)

ART–Antiretroviral Therapy, CD4—cluster of differentiation 4 cells, HIV—Human immunodeficiency virus, RNA—Ribonucleic acid.

### Hospitalization or death

**Fig 2:**
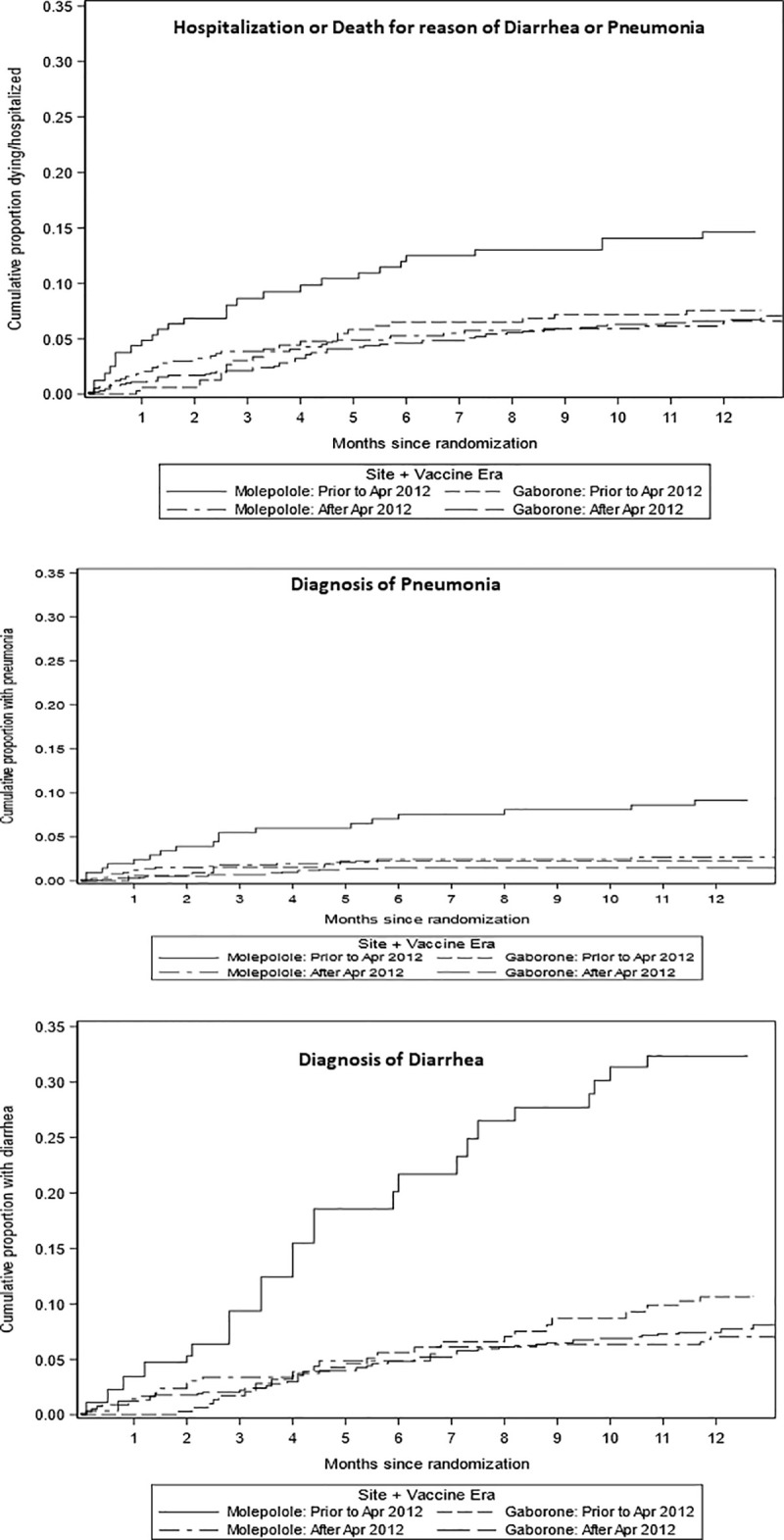
Cumulative proportion hospitalized or dying due to diarrhea and/or pneumonia by time from baseline visit (at age 14–34 days).

Of 279 children with hospitalization or death recorded after the baseline through the 12 months visit, 167 (60%) were either hospitalized or died from causes attributable to diarrhea or pneumonia; 95 were in Gaborone (23 pre-vaccine era vs 72 in the vaccine era) and 72 were in Molepolole (29 pre-vaccine era vs 43 in the vaccine era). The cumulative proportions of infants hospitalized or dying for reason of diarrhea or pneumonia are presented by vaccine era and study site in Fig 2, it demonstrates a marked decrease by vaccine era for Molepolole but less so for Gaborone.

Based on differences observed by study site, the interaction between site and vaccine era was explored in proportional Cox regression models (Table 2). For the outcome of hospitalization or death due to diarrhea or pneumonia, the interaction between site and vaccine era was significant and with similar magnitude of effect, regardless of whether adjustment for additional risk factors was performed.

**Table 2:** Unadjusted and adjusted hazard ratios for selected outcomes related to diarrhea or pneumonia during the vaccine era compared with the pre-vaccine era.

**Outcome**		**Location**		**Difference in HRs between locations: interaction p-value**
		Gaborone	Molepolole	
Hospitalization or death due to pneumonia or diarrhea	Unadjusted HR (95% CI)	0.91 (0.57, 1.45)	0.44 (0.28, 0.71)	P = 0.033
	Adjusted HR (95% CI)	0.91 (0.53, 1.56)	0.46 (0.27, 0.78)	P = 0.067
Diarrhea	Unadjusted HR (95% CI)	0.76 (0.50, 1.15)	0.20 (0.13, 0.29)	P<0.001
	Adjusted HR (95% CI)	0.77 (0.48, 1.24)	0.21 (0.14, 0.32)	P<0.001
Pneumonia	Unadjusted HR (95% CI)	0.69 (0.29, 1.67)	0.30 (0.16, 0.58)	P = 0.14
	Adjusted HR (95% CI)	0.78 (0.30, 2.03)	0.28 (0.13, 0.57)	P = 0.079
First of diarrhea or pneumonia	Unadjusted HR (95% CI)	0.75 (0.51, 1.09)	0.21 (0.15, 0.30)	P<0.001
	Adjusted HR (95% CI)	0.78 (0.51, 1.19)	0.22 (0.15, 0.31)	P<0.001

HR: Hazard ratio for event type shown. CI: Confidence interval. Adjusted HRs are adjusted for gestational age at delivery (<37 vs ≥37 weeks), birth weight (<2500 vs ≥2500g), feeding approach at birth (breastfed vs formula fed), trial randomization arm (cotrimoxazole vs placebo), maternal antiretroviral therapy [ART] prior to delivery (on ART vs not on ART), and maternal CD4 count (continuous variable).

### Diarrhea and pneumonia

We also evaluated the individual diagnoses of grade 3/4 diarrhea and pneumonia, or the combined endpoint of first episode of either events. We observed a decrease in the diagnosis of grade 3/4 diarrhea and pneumonia as single or combined events in the vaccine era, the observed decrease was more marked in Molepolole for diarrhea (HR = 0.20 Molepolole; 0.76 Gaborone), for pneumonia (HR = 0.30 Molepolole, 0.69 Gaborone) and the combined outcome first of either diarrhea and pneumonia (HR = 0.21 Molepolole, 0.75 Gaborone).

Based on differences observed by study site, the interaction between site and vaccine era for these diagnoses was also explored in proportional Cox regression models (Table 2). The interaction between site and vaccine era remained significant for all outcomes other than pneumonia alone. Since there were many more children with diarrhea (111 and 103 overall in Gaborone and Molepolole, respectively) than pneumonia (24 and 36 overall in Gaborone and Molepolole, respectively), precision to identify the difference between locations was reduced for this outcome.

Analysis based on actual vaccine receipt (as-treated analysis) showed similar trends, with decrease in all outcomes noticed for vaccinated infants when compared with those not vaccinated (**S1 Table)**. The rate of decline for all outcomes were still higher in Molepolole as compared to Gaborone, however, observed decline in Gaborone increased and became more discernable with the as-treated analysis (**S1 Fig**). Interaction between site and vaccine receipt remained for all outcomes except the combined outcome of hospitalization and death.

## Discussion

We identified a significant improvement in hospitalization or death from diarrhea or pneumonia, and in diagnoses of grade 3/4 diarrhea and presumed bacteria pneumonia, in HEU children after the rollout of RV and PCV in Botswana. Severe events were lower overall at both study sites after the introduction of both vaccines with greater decline in Molepolole (a rural setting) suggesting greater protection from RV and PCV among HEU in rural locations with higher disease burden.

Many Sub-Saharan African countries have introduced RV and PCV into their national childhood immunization schedule and have reported on remarkable benefits [12]. Within the Sub-Saharan Africa region, studies have consistently shown decline in morbidity and mortality due to both diarrhea and pneumonia following the introduction of these vaccines [15–17]. Where the vaccines were introduced, diarrhea cases have decreased by 10% - 29%; hospitalization from diarrhea by 9% - 43%; and death attributable to diarrhea by 22% across the region [20–24]. Similar outcomes were noted with pneumonia after introduction of the PCV vaccine with declines of 24% in reported cases, 8% - 20.1% in hospitalizations [16, 25], and 12.6% in deaths due to pneumonia [26].

Our observation of higher decline in events in our rural study setting is in accord with prior studies among non-HEU children. A plausible reason for this would be the increased vulnerability of HEU infants to infectious morbidity translating to much higher cases of diarrhea and pneumonia which is further heightened by the environmental and sociodemographic dynamics of a rural setting [27, 28], hence a much larger observed reduction upon vaccination would be expected. More studies are however required to better understand other possible factors that may be contributing to the higher decline observed in the rural setting. Furthermore, our study design focused on grade 3/4 outcomes only, which may have reduced our ability to detect a significant difference between eras in our urban setting where overall events were lower in the pre-vaccine era. Regardless, we believe that decrease in outcomes observed at both study site is attributable to the introduction of both vaccines based on our ability to detect a more noticeable decrease in Gaborone in the as-treated analysis. In addition, most other studies had a longer follow up period (usually 0–59 months), whereas our study reported on findings for the first 12 months of life, a period where children are at increased risk of hospitalization or death due to diarrhea and pneumonia [16, 22]. Therefore, given significant findings for the rural location and a modest trend toward protection in the urban setting, we believe our findings overall are consistent with reports from the region. A study in 2 rural setting in Kenya reported a 57% - 59% reduction in hospitalization due to diarrhea after the introduction of RV [29], while reduction attributable to the introduction of PCV in Soweto South Africa was reported to range from 38.4% to 53.8% [25]. This provides reassurance that these vaccines can provide benefit to the vulnerable HEU population as well.

In our study there were many more children with diarrhea than with pneumonia, or who were hospitalized or died due to diarrhea than pneumonia, giving lower precision to identify difference between locations for pneumonia than for diarrhea. Of note, as in our original analysis [18], the randomized comparison between CTX and placebo did not impact pneumonia or diarrhea results in either study era in the fully adjusted models we considered, receipt of active CTX was not a significant predictor for any of the outcomes.

Our study had several limitations. Temporal confounding between eras was the most important potential confounder. We cannot exclude the possibility that a diarrheal or respiratory outbreak occurred in Molepolole during the pre-vaccine era, which could explain the differences between vaccine eras. However, no such outbreaks were reported, and we do not believe there were differences in diagnosis reporting, likelihood of hospitalization (an outcome determined by independent hospital physicians and not by study staff) or changes in medical care or large social/economic trends that could explain the differences by vaccine era. Also, despite including events occurring 1–2 weeks earlier in the vaccine era due to modification of study eligibility criteria leading to the baseline visit at a younger age, we did not observe more early events in the vaccine era (as would have been expected) and this modification did not appear to impact our analysis. Additional limitations included our inability to determine the main etiology of diarrhea and pneumonia in HEUs. However, we have no reason to believe that etiology differed in HEU children as compared with what has been described in HIV-unexposed children, where *rotavirus* and *S*. *pneumoniae* have been identified as highly prevalent organisms responsible for diarrhea and pneumonia. Finally, our results would have been strengthened with available antibody titer levels to evaluate immunity to *rotavirus* and *S*. *pneumoniae*, but such testing was beyond the scope of the current study and remains a goal for the future.

In conclusion, although temporal confounding cannot be excluded, significant declines in the burden of diarrheal and respiratory illness were observed among HEU children in the most rural location of the Mpepu Study following the introduction of RV and PCV vaccines. These results suggest that RV and PCV may maximally benefit HEU children in rural areas with higher disease burden, and support the broadest possible use of RV and PCV among HEU children to reduce morbidity and mortality in this vulnerable population.

## Supporting information

S1 Checklist CONSORT checklist: Decreased diarrheal and respiratory disease in HIV exposed uninfected children following vaccination with rotavirus and pneumococcal conjugate vaccines. (DOC)

S1 Fig “As–treated” analysis showing unadjusted and adjusted hazard ratios for selected outcomes related to diarrhea or pneumonia during the vaccine era compared with the pre-vaccine era and cumulative proportion hospitalized or dying due to diarrhea and/or pneumonia by time from 4–12 months. (DOCX)

S1 Table Unadjusted and adjusted hazard ratios for selected outcomes related to diarrhea or pneumonia receiving vaccine compared with not receiving. (DOCX)

S1 File (CSV)

S2 File (CSV)

S3 File (PDF)
