# Supplementary material for: Decreased diarrheal and respiratory disease in HIV exposed uninfected children following vaccination with rotavirus and pneumococcal conjugate vaccines
Source: PLoS One. 2020 Dec 21;15(12):e0244100. doi: 10.1371/journal.pone.0244100 (PMC7751865; doi:10.1371/journal.pone.0244100)
Supplement: S1 Table — (DOCX) [file pone.0244100.s003.docx]

| Outcome |  | Location | | Difference in HRs between locations: interaction p-value |
| --- | --- | --- | --- | --- |
|  |  | Gaborone | Molepolole |  |
| Hospitalization or death due to pneumonia or diarrhea^1^ | Unadjusted HR (95% CI) | 0.76 (0.42, 1.37) | 0.57 (0.29, 1.13) | P=0.54 |
|  | Adjusted HR (95% CI) | 0.81 (0.4, 1.64) | 0.69 (0.31, 1.53) | P=0.76 |
| Diarrhea^2^ | Unadjusted HR (95% CI) | 0.50 (0.31, 0.81) | 0.20 (0.11, 0.35) | P=0.01 |
|  | Adjusted HR (95% CI) | 0.52 (0.29, 0.90) | 0.25 (0.13, 0.46) | P=0.07 |
| Pneumonia^3^ | Unadjusted HR (95% CI) | 1.15 (0.32, 4.17) | 0.19 (0.08, 0.48) | P<0.01 |
|  | Adjusted HR (95% CI) | 1.31 (0.34, 5.10) | 0.19 (0.07, 0.53) | P=0.02 |
| First of diarrhea or pneumonia^1^ | Unadjusted HR (95% CI) | 0.57 (0.36, 0.90) | 0.23 (0.15, 0.37) | P=0.01 |
|  | Adjusted HR (95% CI) | 0.64 (0.38, 1.09) | 0.27 (0.16, 0.45) | P=0.02 |

HR: Hazard ratio for event type shown. CI: Confidence interval. Adjusted HRs is adjusted gestational age at delivery (<37 vs ≥37 weeks), birth weight (<2500 vs ≥2500g), feeding approach at birth (breastfed vs formula fed), trial randomization arm (clotrimazole vs placebo), maternal antiretroviral therapy [ART] prior to delivery (on ART vs not on ART), and maternal CD4 count (continuous variable).

^1^Received PCV, RVI or both

^2^Received RVI

^3^Received PCV
