# Supplementary material for: Decreased diarrheal and respiratory disease in HIV exposed uninfected children following vaccination with rotavirus and pneumococcal conjugate vaccines
Source: PLoS One. 2020 Dec 21;15(12):e0244100. doi: 10.1371/journal.pone.0244100 (PMC7751865; doi:10.1371/journal.pone.0244100)
Supplement: S3 File — (PDF) [file pone.0244100.s006.pdf]

BHP Infant Survival Study

A Randomized Study of Cotrimoxazole Prophylaxis and Longer Breastfeeding Duration to Improve Survival among HIV-Exposed Infants in Botswana

Version 2.0, October 12, 2012

A Collaboration of:

The Harvard School of Public Health AIDS Initiative

The Ministry of Health of Botswana

Coordinating Centre:

Botswana-Harvard School of Public Health Partnership for HIV Research and Education

Princess Marina Hospital

Private Bag BO 320, Bontleng

Gaborone, Botswana

Tel: 3902671, Fax: 3901284

Sponsored by:

The National Institute of Child Health and Human Development, NIH

The National Institute of Allergy and Infectious Diseases, NIH

Principal Investigators:	Roger Shapiro and Shahin Lockman
Co-Investigator/Study Coordinators:	Kathleen Powis
	Gbolahan Ajibola
Co-Investigators:	Max Essex
	Michael Hughes
	Joseph Makhema
	Mompati Mmalane
	Kenneth McIntosh
	Anthony Ogwu

Study Team Roster

Principal Investigators

Roger Shapiro, MD, MPH
Division of Infectious Diseases
Beth Israel Deaconess Medical Center
110 Francis St, Suite GB
Boston, MA 02215
Tel: 617-771-0040 (cell)
Fax: 617-632-0766
Email: rshapiro@hsph.harvard.edu

Shahin Lockman, MD, MS
Brigham and Women's Hospital, Boston
Harvard School of Public Health, Boston
Phone: 617-771-8780 (cell)
fax : 617-739-8348
email : slockman@hsph.harvard.edu

Co-Investigators/Study Coordinators

Kathleen Powis, MD
Massachusetts General Hospital
Botswana-Harvard Partnership, Gaborone, Botswana
Phone -- 26774300105
kpowis@partners.org

Nnamdi Ndubuka
Botswana-Harvard Partnership, Gaborone, Botswana
Phone -- 26772115318

Gbolahan Ajibola
Botswana-Harvard Partnership, Gaborone, Botswana
Phone – 26774076767
Email – gajibola@bhp.org.bw

Co-investigators:

M. Essex, DVM, PHD
Harvard School of Public Health, Boston
phone - 001-617-432-2334
fax - 001-617-739-8348
email - messex@hsph.harvard.edu

Michael Hughes, PHD
Harvard School of Public Health, Boston
phone - 001-617-432-2815
fax – 001-617-739-1781
email – mhughes@sdac.harvard.edu

Joseph Makhema, MD
Botswana-Harvard Partnership, Gaborone, Botswana
phone - 3902671
cell phone -
fax - 3901284
email – jmakhema@bhp.org.bw

Mompati Mmalane, MD
Botswana-Harvard Partnership, Gaborone, Botswana
phone - 3902671
cell phone -
fax - 3901284
email – mmmalane@bhp.org.bw

Kenneth McIntosh, MD
Children's Hospital, Boston
Harvard School of Public Health, Boston
phone - 001-617- 355-7621
fax - 001-617-355-8387
email - mcintosh@a1.tch.harvard.edu

Anthony Ogwu, MD
Botswana-Harvard Partnership, Gaborone, Botswana
phone – 3902671
cell phone – 72311139
fax - 3901284
aogwu@bhp.org.bw

Director for Data Operations and IT / Data Management
Erik Widenfelt
Botswana-Harvard Partnership, Gaborone, Botswana
phone - 3902671
cell phone – 71300207
fax - 3901284
email – ewidenfelt@bhp.org.bw

Pharmacist
Tshepho Frank
Botswana-Harvard Partnership, Gaborone, Botswana
tfrank@bhp.org.bw
phone – 3902671

Project Administrator

Ria Madison
Botswana-Harvard Partnership, Gaborone, Botswana
phone – 3902671
cell phone – 72109025
fax – 3901284
email – rmadison@bhp.org.bw

Administrative Assistants

Masego Lewis
Botswana-Harvard Partnership, Gaborone, Botswana
phone - 3902671
cell phone – 72145618
fax - 3901284
email – mlewis@bhp.org.bw

Study Sponsor

Eunice Kennedy Shriver National Institute of Child Health and Human Development
National Institutes of Health

Medical Officers:

Yasaman Shirazi, Ph.D.
Project Officer
Deputy Branch Chief, Pediatric, Adolescent and Maternal AIDS Branch
Eunice Kennedy Shriver National Institute of Child Health and Human Development
National Institutes of Health
6100 Executive Blvd Room 4B11D, MSC 7510
Bethesda Md 20892-7510
301-435-6871
yasaman.shirazi@nih.gov

Lynne M. Mofenson, M.D.
Medical Officer
Chief, Pediatric, Adolescent and Maternal AIDS Branch
Center for Research for Mothers and Children
Eunice Kennedy Shriver National Institute of Child Health and Human Development
National Institutes of Health
6100 Executive Boulevard, Room 4B11
Rockville, MD 20852
Telephone: 301-435-6870
Fax: 301-496-8678

mofensol@exchange.nih.gov or LM65D@nih.gov

Study Management

All questions concerning this protocol, including issues regarding:

Clinical medical management, toxicity management, concomitant medications, laboratory tests, and/or forms development should be sent via e-mail to rshapiro@hsph.harvard.edu or slockman@hsph.harvard.edu

Glossary

ABC	Abacavir
AE	adverse events
ART	antiretroviral therapy or treatment
AUC	area under the curve
BHP	Botswana-Harvard AIDS Initiative Partnership
CBV	Combivir
CK	creatine kinase
CNS	central nervous system
CRF	case report form
CTX	cotrimoxazole
d4T	stavudine
DAIDS	Division of AIDS
ddI	didanosine
EAE	Expedited adverse event (reporting)
EFV	efavirenz
FDA	Food and Drug Administration
HAART	highly active antiretroviral therapy or treatment
IRB	institutional review board
ITT	intent to treat
3TC	lamivudine
LPV	lopinavir
MTCT	mother to child transmission
NNRTI	nonnucleoside reverse transcriptase inhibitor
NRTI	nucleoside reverse transcriptase inhibitor
NVP	nevirapine
PBMC	peripheral blood mononuclear cells
PCR	polymerase chain reaction
PI	protease inhibitor
PTT	partial prothrombin time
QOL	quality of life
RCT	randomized clinical trial
RT	reverse transcriptase
RTV	ritonavir
SD	single dose
TB	tuberculosis
TDF	tenofovir disoproxil fumarate
TZV	Trizivir
ZDV	zidovudine

TABLE OF CONTENTS

1. Study Overview

2. Background and Significance

3. Study Drugs

4. Study Design and Study Sites

5. Inclusion/Exclusion Criteria, and Enrollment of Participants

6. Study Schedules of Evaluations

7. Loss to follow-up, Death, Off-Study

8. Adverse Event Reporting and Toxicity Management

9. Statistical Considerations

10. Human Subjects Considerations

11. Literature Cited

STUDY OVERVIEW

1.1 STUDY SCHEMA

DESIGN

At up to four district hospitals in Botswana, the study will enroll up to 3,724 pregnant or postpartum HIV-1-infected women, and their HIV-uninfected infants, by 4 weeks of infant age. At 14-34 days of age, live HIV-uninfected infants will be randomized to receive either double-blinded CTX or placebo through 15 months. In addition, breastfeeding (BF) infants will be randomized to either BF until 6 or 12 months of age. Children will be followed prospectively until 18 months of age.

The primary endpoint will be survival at 18 months comparing all infants in the CTX vs. placebo arms. Secondary objectives will evaluate HIV-free survival by randomized duration of BF among those BF at randomization; survival and morbidity/mortality at 12 and 15 months; HIV-free survival to 18 months; the safety of CTX prophylaxis comparison of MTCT and mortality by initial feeding method (formula feeding or any BF > 1 month); evaluation of associations between maternal/infant micronutrient deficiency (and humoral/cellular immunity) and subsequent maternal and infant morbidity/mortality; and an analysis of maternal characteristics as predictors for initial feeding choice and HIV-free survival. All HIV-infected women and their infants will receive standard antenatal and intrapartum prophylaxis from the Botswana government for MTCT prevention (PMTCT), and will choose a feeding method with counseling. Breastfeeding, HIV-exposed infants will receive infant nevirapine (NVP) prophylaxis or will be protected from MTCT by the use of maternal HAART.

<u>Figure 1. Diagram of Study Intervention for Randomized Women/Infants</u>

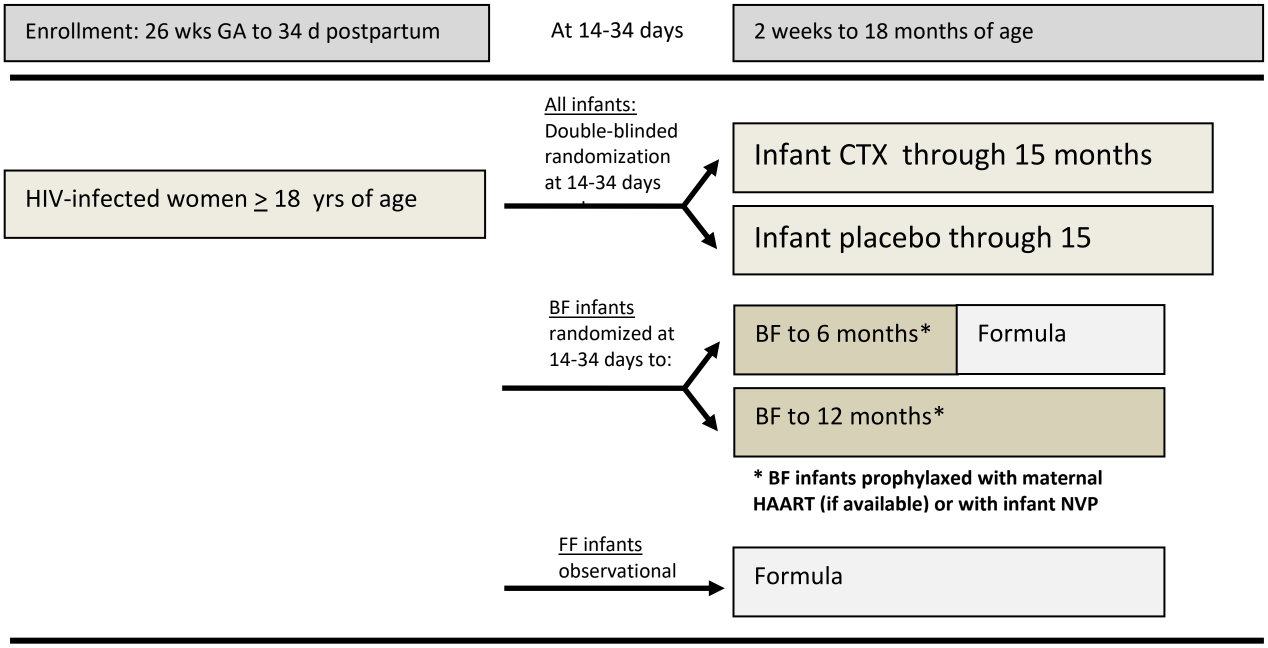

DURATION

Mothers will be followed from the antepartum (or immediate postpartum) period until 18 months postpartum. Children will be followed until 18 months of age.

SAMPLE SIZE

3,724 HIV-infected women will be enrolled between 26 weeks gestation and 34 days postpartum. Surviving HIV-uninfected infants will be enrolled between birth and 34 days of age. HIV-exposed infants eligible for randomization will be randomized between 14-34 days of age.

1.2 Study Steps:

Step 1: SCREENING FOR HIV IN ANTENATAL CLINICS

- Screen for HIV per government protocol
- Post-test counsel at government clinic sites

Step 2: ENROLLMENT OF WOMEN/INFANTS

- Introduce study to all women from 26 weeks gestation to 34 days postpartum

<u>For women choosing to participate in study</u>

- Sign study consent
- Feeding counseling
- Enroll infants between birth and 34 days

Step 3: RANDOMIZATION OF HIV-EXPOSED INFANTS AT STUDY CLINIC SITES (Day 14-34)

- For all HIV-exposed infants: CTX vs. placebo randomization
- For HIV-exposed, BF infants: Infant 6 vs. 12 months breastfeeding randomization

Step 4: INFANT FOLLOW-UP AT STUDY CLINIC SITES TO 18 MONTHS

- Feeding intervention ends at 6 or 12 months (HIV-exposed infants)
- CTX vs. placebo intervention ends at 15 months (HIV-unexposed infants)
- Off-study at 18 months (all infants)

1.3 HYPOTHESES AND OBJECTIVES

Primary Objective:

- To compare overall survival from randomization (at 14-34 days of age) to 18 months of age among HIV-exposed/uninfected infants who are randomized to receive CTX vs. placebo in this period. *[Hypothesis: Survival rates will be higher in the CTX arm].*

Secondary CTX Objectives:

All HIV-Exposed/Uninfected Infants

- To compare hematology profiles, morbidity, hospitalization, and adverse events from randomization through 18 months of age among infants randomized to CTX vs. placebo *[Hypothesis: CTX will be associated with more infant neutropenias and possibly anemias; however, these will be clinically insignificant and there will be no significant difference at 18 months of age. CTX will reduce morbidity and hospitalization events through 18 months of age.]*
- To compare toxicity from randomization through 18 months of age among infants randomized to CTX vs. placebo, by maternal HAART exposure *[Hypothesis: Additive toxicity from maternal HAART exposure from BF plus CTX will not exceed that of maternal HAART exposure plus placebo].*
- To compare *HIV-free* survival from randomization to 18 months among infants randomized to receive CTX vs. placebo. *[Hypothesis: HIV-free survival rates will be higher in the CTX arm].*
- To explore survival and safety differences among infants randomized to CTX and placebo by initial chosen feeding method (FF vs. BF) and by randomized feeding arm if BF *[Hypothesis: there will be no difference in the magnitude or direction of outcomes with CTX vs. placebo by feeding method]*
- To perform an exploratory analysis of CTX resistance in clinically significant respiratory and diarrheal pathogens identified from infants in the study *[Hypothesis: CTX resistance will be higher among specimens from the CTX than the placebo arm]*

Secondary Feeding Objectives:

All HIV-Exposed/Uninfected Infants

- To evaluate survival and MTCT among infants born to women who enroll in the study in the antenatal period, by infant feeding method chosen by mother (FF vs. BF) *[Hypothesis: Mortality and MTCT rates will be similar to those seen in previous studies].*
- To evaluate potential predictors of adverse outcomes with FF or BF from birth based on an initial AFASS assessment. *[Hypothesis: even when infant feeding counseling is provided, it will be possible to identify factors that predict whether it is safe for an individual HIV-infected mother to FF].*
- To explore survival and safety differences for all feeding groups by CTX/placebo randomization group *[Hypothesis: there will be no difference in the magnitude or direction of outcomes by CTX/placebo receipt].*

Randomized BF Infants

- To compare HIV-free survival at 18 months among infants who are randomized to BF until 6 months of age vs. BF until 12 of age months, among infants of mothers who choose to BF. *[Hypothesis: HIV-free survival rates will be higher in the 12-month arm].*
- To compare HIV-free survival at 18 months of age by the actual duration of BF (as treated) *[Hypothesis: HIV-free survival will be higher among those BF for a longer duration.*
- To compare overall survival, morbidity, and growth parameters by feeding arm and by the actual duration of BF (as treated) *[Hypothesis: Survival will be higher, and morbidity lower among those BF for longer duration].*
- To compare MTCT rates at 3, 6, 9, 12, 15, and 18 months by actual feeding method through each time point *[Hypothesis: MTCT rates will be low and comparable to the Mashi Study at 4 weeks, and low rates of late MTCT will occur among infants prophylaxed with either NVP or maternal HAART while BF through 6 months and 12 months].*

Other Secondary Objectives:

- To determine whether maternal HLA type or other maternal markers predict HIV-1 viral set-point, or risk for MTCT, among HIV-infected women.
- To assess the relationship between maternal/infant micronutrient levels/immune function and infant morbidity/mortality in HIV-infected women and their HIV-uninfected infants.
- To describe morbidity and mortality and the timing of treatment with antiretrovirals among infants who become HIV-infected during the study.
- To assess factors associated with favorable and unfavorable infant growth patterns

2. BACKGROUND AND SIGNIFICANCE

2.1. General Rationale for Use of CTX Prophylaxis to Reduce Mortality and Morbidity among HIV-Uninfected Infants

As improved MTCT prevention interventions reduce the number of HIV-infected infants in the antepartum and peripartum periods, interventions to improve HIV-free survival among HIV-uninfected infants are needed. Morbidity and mortality are increased among HIV-uninfected infants born to HIV-infected mothers [1-4], and reduced infant survival among HIV-exposed infants may lead to as many deaths as HIV infection itself. In Botswana, the use of formula feeding or shorter breastfeeding may worsen the problem of early infant mortality among HIV-exposed infants. The risk associated with formula has been demonstrated in the Mashi Study [5] and in a large diarrheal disease outbreak in 2005-2006 [6]. Studies in Botswana and elsewhere also have demonstrated high infant mortality rates after weaning from breast milk at or before 6 months [5, 7-8], as evidenced by "catch up" mortality that occurred in the BF arm of the Mashi Study after 6 months. In the Mashi study, 1200 HIV-infected pregnant women were randomized to either breastfeed with prolonged (6-month) infant zidovudine prophylaxis (and weaning to formula and foods at 6 months of age), or to formula feed from birth with 1 month of infant zidovudine (women/infected infants gained access to HAART partway through the trial). Cumulative mortality – even *among HIV-negative infants* – was higher than among children born to HIV-negative mothers, and did not differ by feeding arm at 12 or 18 months [5, 9]. The period after weaning (or early in life if formula feeding) appears to be the greatest period of risk, and it is not known whether a longer period of breastfeeding would reduce mortality.

CTX has demonstrated a mortality benefit among HIV-infected infants, but no randomized studies to date have evaluated prophylactic CTX to reduce infant mortality among HIV-uninfected infants at weaning. Feeding strategies minimizing late MTCT risk (with FF or with a limited period of BF) may be possible for large areas of the developing world if supported by CTX prophylaxis. We hypothesize that among HIV-uninfected children born to HIV-infected mothers, the mortality risk associated with FF and with early weaning may be mitigated by the prophylactic use of infant CTX.

2.2. High Mortality Risk with FF and After Early Weaning Among HIV-Exposed Infants
Recent study data from Africa identify two groups of infants with particularly high mortality: 1) data from the Mashi Study demonstrate 5.9% mortality in the first 3 months of life among infants in the formula arm, compared with 2.4% in the breastfed arm [10], and 2) data from Mashi as well as other African studies have confirmed high infant mortality after weaning from breast milk (especially at 4-6 months or earlier) [5, 8, 11]. These two risk periods may in fact be combined under one potential explanation: greatest mortality risk may occur in the first period of life when an infant is not breastfed. After the first 3 months of life, formula fed infants in Mashi had a *lower overall mortality rate* than the breastfed infants: 4.8% vs. 6.3% through 24 months (Figure 2), and only a small amount of this mortality could be attributed to infant HIV infection in the BF arm.

Most of the “catch-up” mortality in the breastfed arm occurred between 6-12 months, after weaning (3.2% of children in the breastfed arm vs. 1.3% of children in the formula arm died between 6-12 months). No study has addressed whether the weaning risk differs by infant age during the first year of life. Data from the pre-HIV era suggest that 12 months of breastfeeding improves infant survival [12]. It is unknown how long to recommend breastfeeding for HIV-exposed infants in a setting where formula is available and potentially safe, or where maternal HAART or extended NVP prophylaxis are available for infants. This recommendation depends upon the ongoing risk of MTCT vs. the unknown risk of mortality after weaning at different ages. Given the data from the Mashi study and other studies, it is unknown whether this “weaning risk” – or the risk of mortality in the first period of life when breast milk is not given – differs between the first few months of life vs. the sixth month of life vs. 1 year of life. New WHO recommendations for PMTCT and infant feeding were released in 2010, and recommend extended maternal HAART or infant NVP for a 12 month period. Whether this recommendation is applicable or appropriate for Botswana remains unknown.

Figure 2. Mortality Risk among Formula-fed and Breastfed Infants, Mashi Study, Botswana

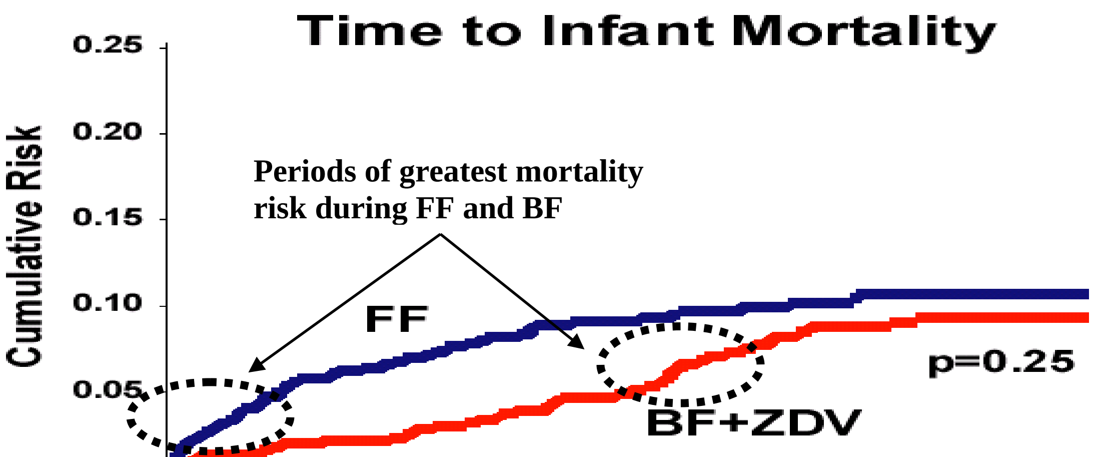

2.3. Cotrimoxazole Reduces Morbidity and Mortality in HIV-Infected Populations.
Cotrimoxazole is an inexpensive and safe drug used to prevent *Pneumocystis jiroveci* pneumonia (PCP) and other infections among HIV-infected individuals.

Its protection extends beyond opportunistic infections, and includes many pathogens that cause pneumonia and diarrhea during infancy [13-15]. CTX is active against a wide range of gram positive and gram-negative bacterial pathogens, and it is a preferred agent whenever bacterial prophylaxis is considered in clinical settings. Importantly, it is already the recommended agent used to prevent PCP in HIV-infected infants or in HIV-exposed infants with an unknown status, and it is therefore widely available for distribution to HIV-exposed infants throughout Africa.

The benefits of CTX have been demonstrated among adults and children with HIV infection, including settings with a high prevalence of bacterial pathogen resistance to CTX [15-16]. In Abidjan, a 46% mortality reduction was noted in the CTX arm compared with placebo ($P<0.001$) [13]. In Uganda, where CTX resistance to diarrheal pathogens is high, a 46% mortality reduction ($P=0.006$) was also noted after CTX was initiated in a longitudinally followed cohort [15]. Among HIV-infected infants in Zambia, a 43% mortality reduction was noted between CTX and placebo ($P=0.0002$) [16]. In Malawi, 41% mortality reduction was noted among HIV-infected adults within 6 months of starting HAART [17]. In Tanzania, significantly fewer deaths occurred among HIV-infected infants and children receiving CTX [18]. In both Zambia and Tanzania, the benefit of CTX was *independent of CD4 cell count %*, suggesting potential applicability to HIV-uninfected infants.

2.3.1 Rationale for CTX in HIV-Exposed but Uninfected Infants.
We hypothesize that CTX could improve survival among vulnerable HIV-exposed but uninfected infants in Botswana. We know from the Mashi Study (where infants did NOT routinely receive CTX if they were HIV PCR negative) that the primary cause of infant deaths among HIV-exposed uninfected infants was from respiratory and diarrheal illnesses [9], and this has been demonstrated elsewhere in Africa as well [11, 19-21]. Whether or not CTX prophylaxis has a positive impact on overall survival among HIV-exposed but uninfected children has not been directly studied in a randomized trial.

CTX was chosen as our prophylactic agent for several reasons: 1) it is the only prophylactic agent with proven all-cause mortality benefit for HIV-infected children, and no trials exist to guide use of any other agent among HIV-exposed children; 2) it has broad efficacy against a wide range of bacterial and parasitic pathogens; 3) it is unreliable as a treatment agent and

already widely used for prophylaxis among HIV-infected or HIV-unknown infants, which somewhat lessens resistance concerns; 4) there is an established safety record for use as infant prophylaxis, and established dosing guidelines.

The CHAP Study from Zambia provides support for the hypothesis that CTX will have efficacy among HIV-exposed but uninfected infants. This study was a randomized trial among HIV-*infected* children aged 1-14, and it was stopped early for a 43% mortality reduction in the CTX arm [16]. In this study area, CTX resistance among bacterial pathogens was known to be high (60-80%). PCP infection was not detected in any nasopharyngeal swabs in the study, but more overall pneumonias were reported in the placebo arm than in the CTX arm. In addition, similar mortality reductions were found for infants with higher and lower CD4 percentages (i.e., benefit was documented among infants at lower risk for opportunistic infections). This suggests that the benefit was because of a prophylactic effect against standard bacterial pathogens (presumably CTX resistant *in vitro*) that cause diarrhea and pneumonia, even when resistance is common. The authors of the CHAP Study concluded that *in vitro* resistance testing may not be an indication of prophylactic efficacy. These study results have recently been used to demonstrate the cost-effectiveness of CTX for HIV-infected children [22].

A recently reported study among HIV-infected children in Tanzania showed similar findings [18], even in the setting of very small numbers. Among 120 children between 5 months to 13 years, diarrhea was almost 3 times as common in those not receiving CTX ($P<0.001$), and cryptosporidium (a common cause of diarrhea in all children) was significantly less common in those receiving CTX ($P=0.001$). CTX was also significantly associated with less fever and less use of other antibiotics. CD4 cell count < 15% was *not* associated with death ($P=0.76$), but lack of CTX was the *strongest predictor of mortality in the study*: of 13 deaths, 12 were among infants not receiving CTX ($P<0.001$). The lack of association between CD4 cell count and mortality, but strong association with CTX, is encouraging for generalizability of these results to HIV-exposed but uninfected infants.

Other studies of CTX among HIV-infected individuals have shown an effect for pathogens common to both HIV-infected and uninfected persons. Among 545 HIV-infected adults enrolled in a study in Abidjan, the most significant difference between CTX and placebo was noted for bacterial pneumonias ($P=0.0009$). Significant differences in the number of positive blood cultures were noted between arms [14]. In another Abidjan study of HIV-infected adults, hospital admission for all diseases that could potentially have been prevented by CTX prophylaxis (septicemia, enteritis, chest infection, urinary-tract infection, and toxoplasmosis) was significantly lower in the CTX arm than in the placebo arm (hazard ratio 0.3, $P=0.001$) [13]. In a recent study from Uganda, the risk of diarrhea among HIV-infected adults who discontinued CTX after improved CD4 cell counts on HAART increased 1.8-fold ($P<0.0001$) compared with those remaining on CTX [23].

There are other data which also support the potential efficacy of CTX prophylaxis among HIV-uninfected individuals. Benefit was demonstrated among HIV-uninfected household *contacts* of HIV-infected patients who were receiving CTX in Uganda; among HIV-uninfected pediatric contacts (not taking CTX themselves), significant reductions were seen in mortality (HR 0.37), malaria (HR 0.65), diarrhea (HR 0.58), and hospitalizations (0.53) [24]. Because this study occurred in a region with endemic malaria, it is unknown how much of the benefit was from

malaria reduction in the household, or from improved health in caregivers. It is therefore unknown how applicable these findings are for Botswana (malaria is not present in our study locations). In one observational study from South Africa, CTX receipt was associated with fewer lower respiratory tract infections among HIV-exposed but uninfected infants [25]. Although it is believed that the potential beneficial effect of CTX is from prevention of routine bacterial infections, PCP has also been described among HIV-exposed infants [26], and CTX would be expected to dramatically reduce any associated mortality from PCP in these infants. The potential contribution of PCP to mortality among HIV-exposed but uninfected infants is unknown.

2.3.2 Vulnerability of HIV-Exposed Infants with Limited BF.
Because of the success of CTX in Cote d'Ivoire, Uganda, and Zambia, and because of the particular vulnerability of HIV-exposed infants to common bacterial infections (and perhaps even to PCP), we hypothesize that CTX may improve infant survival. We also believe that it may be especially important among infants who are weaned early (by 6 or even 12 months of age) from breast milk and do not yet have a fully mature immune system. These infants may in fact be as vulnerable to bacterial infections as the HIV-infected children and adults studied in the previous CTX trials – the high 1-year mortality rates from HIV-exposed but *uninfected* infants in the Mashi Study (7-8%) and other studies is similar to the annual mortality risk for HIV-infected adults and older children. We are unaware of any other planned study to evaluate CTX vs. placebo during this early high-risk period among infants formula-fed from birth or among infants who wean early from breastfeeding. We believe the findings from this study will be broadly applicable for both FF and BF populations, and among populations that wean from BF in the first year of life; most infants in the study are expected to breastfeed for either 6 or 12 months. Thus our study will have applicability for both formula-fed and breastfed populations.

We believe that our evaluation of CTX from 4 weeks to 15 months of life maximizes the public health utility of this potential intervention, and maximizes our ability to detect a true biological effect of CTX. The first year of life is the most vulnerable for both HIV-infected and HIV-exposed, uninfected infants, and the time when we observed the steepest mortality rates in the Mashi Study. HIV-exposed infants may have increased vulnerability in the first year of life based on their high rates of early mortality (see Section B2), and protecting infants during this period may be of greater importance than doing so at a time when their own immune systems may be better able to fight infections. As discussed previously, the optimal age for weaning of an HIV-exposed infant remains unknown, but this study will have broad applicability for the large number of at-risk infants who wean at up to 1 year of life (per new WHO guidelines) or FF from birth. Of importance, the strategy is consistent with the use of CTX for HIV-infected infants, and fits with programmatic goals to maximize infant survival while minimizing the amount of time that women and infants need to remain on prophylactic ARVs (for those receiving ARVs for PMTCT only) and on CTX.

2.3.3 CTX is Underutilized in HIV-Exposed Infants.
Scale–up of CTX programs for HIV-exposed infants with unknown HIV status has been advocated by WHO and others to reduce mortality among HIV-infected infants [27-28]. It is estimated that only 4% of infants who meet WHO criteria for CTX currently receive it [28]. The use of CTX for HIV-infected (or potentially infected) infants is viewed by WHO and others as a critical intervention that needs to be prioritized at the country level [27]. Without data from a

randomized trial to inform about the benefit (or lack thereof) of CTX for HIV-exposed but *uninfected* infants, it remains difficult for public health programs to estimate the potential lives saved by implementing CTX. If CTX were shown to be of value to *all* HIV-exposed infants – including those who remain HIV-uninfected -- this would strengthen support for the recommendation for its use in all circumstances, whether the infant HIV status is known or unknown. Although such support should exist currently when HIV-status is unknown, the belief that CTX only benefits the few HIV-infected infants (who may have a limited lifespan in regions without HAART access) has limited enthusiasm for the widespread rollout of CTX. Further, for programs such as Botswana's where dried blood spot PCR testing is being implemented, studying CTX among documented HIV-*uninfected* infants is directly relevant to recommendations for this population. As PCR testing becomes more widely available, more and more programs will have to decide whether to recommend CTX for those infants testing negative. This study will provide the needed information.

2.3.4. CTX Dosing

The dose of CTX that we propose to use is the same as the standard prophylactic dose for HIV-infected infants (other than for infants 2-4 weeks of age; please see Section 6.1 below for details on dosing). Although we would ideally like to begin CTX as soon as possible after birth, CTX may be unsafe among infants less than 2 weeks of life because of possible hyperbilirubinemia among preterm or possibly preterm infants, and its dosing may be complicated by slower metabolism of sulfa drugs in the immediate postnatal period. The rationale and potential risks and benefits of initiating study drug at 2 weeks is described in detail in Section 10 below.

2.3.5. Potential Risks of Infant CTX Prophylaxis

We believe that the potential benefits of the interventions in this study outweigh the risks. The primary risks are the possibility of stigma, unsafe use of formula, early MTCT, potential toxicity from or increased resistance to NVP and CTX, and PCP among HIV-infected infants before CTX is started. As described above, we have data from the Mashi Study to support a low risk for early MTCT in a setting of SD NVP and ZDV prophylaxis. Extended NVP has been studied in this same context in previous trials [29-30] without evidence of added toxicity, and CTX is used extensively and safely among HIV-exposed infants throughout Africa per WHO recommendations. CTX is already provided to HIV-exposed BF infants, (and HIV-exposed FF infants without a definitive HIV status by PCR testing) throughout the world per WHO guidelines, and the pros and cons of the resistance implications of this policy have been well-described [27]. Infants who become HIV-infected during breastfeeding may be randomized to the placebo arm and not receive CTX until the HIV infection is recognized. We have taken extensive measures to minimize the risk of this among the few infants who may become HIV-infected during BF with maternal HAART prophylaxis. For a complete discussion of toxicity, resistance, and CTX coverage concerns, please see Section 10.

2.4. Infant Feeding Considerations

In the proposed study, in the setting of a FF-based national program in Botswana and the potential for AFASS conditions to be met by participants, we believe it is reasonable to recommend to mothers feeding options that have been proven to offer maximal MTCT prevention. These include exclusive BF + infant NVP, exclusive BF + maternal HAART, or exclusive FF (if this is deemed to be AFASS for an individual participant). We will therefore offer enrollment and support to all otherwise eligible HIV-infected mothers and their infants

regardless of the feeding method that is ultimately chosen by the mother after counseling and discussion.

Because we do not know the most appropriate time to wean from BF in the setting of MTCT prophylaxis to maximize HIV-free survival, a second randomization will occur concurrently with the CTX/placebo randomization (also balanced by clinical site) for infants whose mothers choose to BF. These infants will be randomized to either 6 months or 12 months of BF, with ongoing MTCT prophylaxis. All infants who are being BF at the time of randomization to CTX/placebo will be randomized to strategies that encourage either 6 vs. 12 months of BF, regardless of the intended duration of BF at the time of randomization. Please see Section 8 for considerations related to actual duration of BF. Although feeding recommendations differ depending on local conditions – ranging from complete BF avoidance in the developed world to longer BF where AFASS conditions are not met (or where replacement feeding is not available) – recent data for PMTCT strategies using maternal HAART or infant NVP are likely to at least bring equipoise to this decision process for all areas in Botswana (or southern Africa) where this study would occur, and may tip the balance to favor BF with maternal or infant prophylaxis for most women.

The CTX intervention is aimed at supporting infant survival in areas where limited BF commonly occurs. We hypothesize that the risks from FF or after early weaning may be mitigated by the prophylactic use of CTX, and that feeding strategies minimizing late MTCT risk may be possible for large areas of the developing world if supported by CTX prophylaxis. The CTX randomization is expected to be equally effective for either formula-feeding or breastfeeding populations, and at different weaning ages within the first year of life.

2.4.1. MTCT Risk in Breastfed infants (with Infant Prophylaxis or Maternal HAART)

Several studies now suggest that minimal MTCT occurs early in life in the setting of BF + infant antiretroviral prophylaxis, or among infants whose mothers are receiving HAART. During the first month of life, the Mashi Study detected essentially no transmissions from breastfeeding with use of maternal antepartum ZDV (and HAART for women with AIDS later in the study), single-dose NVP vs. placebo in mothers and infants (changed to all active NVP in infants in the later half of the study), and a full month of infant ZDV [31]. Recent studies elsewhere in Africa have demonstrated the efficacy and safety of infant NVP prophylaxis during BF [29, 32], and these data have now been extended out to at least 6 months of age with the BAN Study [44]. In the SWEN and the PEPI-Malawi studies, MTCT rates using extended NVP prophylaxis were significantly lower than the single-dose NVP control arm especially during the period of the intervention (6 weeks in SWEN, 14 weeks in PEPI-Malawi). In the PEPI-Malawi study, between 6 and 14 weeks of age, 1.1% of previously HIV-negative children became infected. In the BAN Study, 1.8% of infants became infected between birth and 6 months when receiving extended NVP. This small amount of transmission may be lower than the additional mortality that would occur from FF (in Mashi FF infants, 3.4% died from birth to 1 month, and 5.9% died through 3 months). Furthermore, one would expect postnatal MTCT rates with infant NVP prophylaxis to be even lower among the subset of women who have higher CD4 cell counts (in the proposed study, those with lower CD4 cell counts will receive HAART). Data from the Botswana Mma Bana Study and from other African studies suggest that extremely low BF MTCT rates occur among women receiving HAART through at least 6 months postpartum. In Mma Bana, only 2 late transmissions occurred among 709 live borne infants (0.3%). In the AMATA and Mitra Plus Studies, MTCT rates were 0.6% and 1.1%, respectively, through 6 months among infants who

were uninfected at birth [33-34]. In Botswana, pregnant women with CD4 < 250 cells/mm^3 are eligible to receive HAART.

Because of the data summarized above, we believe that it is reasonable to support the options of BF + extended infant NVP for HIV-infected mothers not taking HAART, and BF + maternal HAART for women who are receiving HAART. These strategies are in accord with WHO PMTCT recommendations. We believe – as do others in the international community and at the WHO – that at this time a distinction between maternal HAART and infant prophylaxis cannot be made through 6 months (and probably 12 months). Although these interventions have not been studied from 6-12 months, they are expected to prevent MTCT in this period and the benefits may exceed the risks for many women. By studying 6 vs. 12 months of BF, we hope to inform Botswana PMTCT policy. The current Botswana MTCT program offers free infant formula to HIV-infected women for up to 12 months, and we will also allow FF from birth as an option for women for whom FF is deemed to be AFASS and who choose to FF. From a cost perspective, infant formula provision during the first 12 months of life is likely to be more expensive than NVP prophylaxis, and similar to the cost of maternal HAART.

2.4.2. How Long Should HIV-Exposed (but Uninfected) Children Breastfeed?
As discussed above, the optimal age of weaning is unknown. The risk of late MTCT during ongoing breastfeeding (without maternal HAART or infant NVP) was 4.4% between months 1-7 in Mashi and has been higher in other studies. In the SWEN and PEPI-Malawi studies, BF MTCT increased substantially with longer BF *beyond the NVP intervention periods* (8.4% from 14 weeks to 24 months in PEPI-Malawi, 4.4% between 6 weeks and 6 months in SWEN). Longer BF is likely to reduce infant mortality, but the balance with HIV-transmission is unknown. In the absence of prophylaxis, the ZEBS study showed equivalent HIV-free survival for 4 months of breastfeeding vs. up to 18 months of breastfeeding, but HIV infection made up a substantial percentage of the outcomes in both groups and there was substantial cross-over [11]. In Mashi, infant feeding did not predict overall mortality (or HIV-free survival) at 24 months. A 6 month BF strategy may offer a better alternative for those women who need to return to the workforce and cannot BF for a long period, or for women/programs wanting to minimize late MTCT to the greatest extent possible, and where FF after 6 months may be AFASS. However, 12 months of BF may improve overall HIV-free infant survival and avoids the need for any formula use as infants can be weaned to “the family pot” at 1 year. Pre-HIV era data suggest that 6 months of BF is sufficient to provide most of the nutritional and immunologic benefits of BF [12, 35-37], although survival was improved in many settings by BF for at least a 12 months [12]. We have experience weaning at 6 months in the Mma Bana and Mashi studies and we know that virtually all women are willing and able to wean at this time. We expect women to be willing to wean at 12 months in the same manner.

2.4.3. Is Formula Feeding a Reasonable Option in Resource Limited Settings?
Limited BF is already common in many areas of the developing world. HIV-exposed infants are generally formula fed from birth in Botswana, and in urban areas of South Africa, West Africa and East Africa [38-40]. Breastfeeding may be limited because women are unable to breastfeed, and many women in Botswana and other middle-income nations choose either to formula feed from birth or to wean early because they must return to work (generally before 3 months postpartum).

Botswana surveillance data suggest that ~ 90% of HIV-infected women currently choose to formula feed based on government recommendations (although 6 months of BF in the context of our PMTCT Studies has been highly acceptable when available). In South Africa, more than 20% of all woman are not breastfeeding by 2 months [41], regardless of HIV status. Formula feeding "if conditions for safe and adequate formula feeding are possible and highly probable" is recommended for HIV-exposed infants in South Africa [42], and at most urban and periurban centers HIV-infected women are counseled to formula feed from birth. For example, approximately 96% of women in Soweto and in the Western Cape (including Cape Town) formula feed from birth (Dr. James McIntyre, personal communication, 2008). The last national PMTCT evaluation in South Africa was in 2002, and 58% of all HIV-infected women chose to formula feed and 42% to breastfeed; feeding policy has not changed since this time, and these numbers are believed to remain accurate (T. Doherty, personal communication, 2008). Because of South Africa's large population and high HIV prevalence, this represents a very large percentage of the overall feeding choices being made by HIV-infected women in Africa.

Mashi Study data demonstrate identical long-term survival and HIV-free survival among FF vs. BF at these same clinical sites in Botswana, and we therefore plan to support FF for women who can do so safely. *Because the Botswana government policy currently supports the option of FF from birth, we believe it is important to include this option for women who choose it.* A benefit of including women who FF from birth is that it expands the generalizability of the study.

2.4.4 Anticipated Feeding Choices Among Study Participants

The success of the BF intervention will depend upon the final number of women who choose to BF. As originally designed, we anticipated the following approximate breakdown of maternal feeding choices in this study: 25% of women on HAART, and the majority of these women will choose to BF; of the remaining 75% of women who are not receiving HAART, 75% will choose exclusive BF + NVP and the remaining women will choose to exclusively FF. We therefore anticipated ~ 2,679 total breastfeeding women in this study: ~827 (25% of 3,308) enrolled will choose to BF (with maternal HAART), and ~ 1,852 (75% of the 2,481 not receiving HAART) will choose BF+NVP. However, these calculations assumed a shift to BF + prophylaxis (infant NVP or maternal HAART) in government guidelines which had not occurred by the end of 2011. Therefore, the final distribution of FF vs. BF in the study remains unknown, and may depend upon the training and practices that occur after the release of updated government feeding guidelines. Feeding decisions will be closely monitored in the study, including by the Data and Safety Monitoring Board. The study team recognizes that objectives related to the BF randomization may be underpowered, and exploratory in nature, if fewer women BF than previously anticipated.

2.4.5. Use of AFASS Criteria to Make Initial Feeding Choices and to Predict Outcomes

The Good Start study in South Africa is the only study that has shown feeding choices to affect MTCT or mortality outcomes at an individual level [43]. In the Good Start Study, AFASS criteria were identified by a simple screen that included: "piped water in the house or yard (safety); electricity, gas or paraffin for cooking fuel (feasibility); disclosure of HIV status by 3 weeks after birth (acceptability); having someone in the household employed (affordability and sustainability); and access to a fridge for storage of prepared formula (safety)." FF infants whose mothers had piped water, a fuel source, and had disclosed their HIV status had a significantly improved HIV-free survival than other FF infants. However, residual confounding by study site

was a potential problem in this study, as most poor FF outcomes occurred at the site with the lowest socioeconomic status (SES) indicators.

In Botswana, a similar analysis could be performed within a more homogeneous population with more similar SES indicators. Further, the specific criteria used to screen for AFASS are local in nature, and validation of criteria used in South Africa (with modifications appropriate to conditions in Botswana, including the availability of government-supplied formula) needs to occur. The study design allows for prospective evaluation of whether or not mothers (with counseling) make appropriate initial feeding decisions (FF vs. BF) with regard to AFASS criteria, and determination of specific AFASS criteria that predict which infants can and cannot safely formula feed in Botswana.

2.4.6. Micronutrients and Innate Immunity

Studies from Tanzania have identified two potentially modifiable risk factors contributing to increased mortality, poor growth and low vitamin D levels among HIV-exposed/uninfected infants. In Botswana, we have also identified associations between decreased early growth among HIV-exposed/uninfected infants and *in utero* HAART exposure, and a possible reversal of this pattern during breastfeeding. It is therefore important to evaluate both Vitamin D levels and growth patterns and their associations with (1) infant morbidity/mortality, and (2) *in utero* and breast milk HAART exposure. Other potential explanations for increased susceptibility to infection include insufficient innate and adaptive immunity. As innate immunity directs the subsequent adaptive immune response, it may affect infectious morbidity and mortality in HIV exposed but uninfected infants. Further, it is possible that CTX may alter immune function independent of the antimicrobial response, and this possible effect will be explored by evaluating markers of immune function in each study arm.

3. STUDY DRUGS

3.1 CTX

CTX is a combination antibiotic of trimethoprim and sulfamethoxazole used in the treatment and prophylaxis of a variety of bacterial infections.

CTX is the most common antibiotic used in African infants, and it may be associated with a small risk of neutropenia and anemia. CTX is recommended by the World Health Organization for use as prophylaxis against PCP among all HIV-exposed infants who are breastfeeding or who do not have access to PCR testing to confirm their HIV status, so it is used widely throughout Africa as prophylaxis in infants.

The most common side effects are gastrointestinal (nausea, vomiting, diarrhea). Rash and fever are rare but reported side effects in children. Use of CTX also may result in marrow suppression which could lead to neutropenia and anemia. The prevalence of side effects appears to be low. Among 395 HIV-exposed Thai infants who received CTX prophylaxis from 6 weeks of age through 12 months, seven children (1.8%) experienced adverse events (5 rashes, 1 oral ulcer, 1 mild anemia), and all improved after prophylaxis was stopped. In a Zambian study of HIV-infected children, there was no difference between randomized treatment and control groups in

the incidence of one or more grade 3 or 4 adverse drug reactions (HR 0.76; 95% CI 0.39-1.5) [16].

CTX also carries a low risk for contributing to kernicterus when used in the first month of life, although clinical and epidemiological data for this are lacking; because of this theoretical risk, CTX will not be used until at least 2 weeks of life (4 weeks in those born < 36 week gestation). Although the World Health Organization recommends prophylaxis in HIV-exposed infants starting as early as 4 weeks, treatment cohorts and safety data provide strong evidence that starting at 2 weeks poses little or no kernicterus risk. However, this earlier start expands the intervention to potentially prevent additional mortality; based on Mashi Study data, more than 15% of all FF infants who die may do so between 2 and 4 weeks of life. To minimize the potential for toxicity and harm with this earlier start date, all infants will receive NVP prophylaxis in the first month (rather than ZDV) to reduce the risk of additive marrow toxicity; weight-based and gestational age-based dosing will occur; and a clinical assessment for jaundice will occur before starting study drug from 14-28 days. A complete discussion of the risks/benefits of starting CTX from 14-34 days (rather than 28-34 days) is presented in Section 10.

3.2 NVP

NVP is an NNRTI with activity against HIV-1 and is structurally a member of the dipyridodiazepinone chemical class of compounds. NVP binds directly to RT and blocks the RNA-dependent and DNA-dependent DNA polymerase activities by causing disruption of the enzymes' catalytic site. The activity of NVP does not compete with template or nucleoside triphosphates.

The safety of NVP has been assessed in more than 2800 patients in clinical trials. The experience from clinical trials and clinical practice has shown that the most serious AEs are clinical hepatitis/hepatic failure, Stevens-Johnson syndrome, toxic epidermal necrolysis, and hypersensitivity reactions characterized by rash, constitutional findings, and organ dysfunction. Fatalities due to these serious AEs have been reported.

However, adverse events from the use of infant prophylactic NVP are rare. NVP has been used for 6-14 weeks of ongoing infant prophylaxis in 2 recent international clinical trials without significant toxicity. In the PEPI-Malawi Study, SAEs were not significantly increased between the arms that received extended NVP vs. single-dose NVP at birth (N=609) [29]. In the SWEN Study, there was also no difference in SAEs for these comparator groups (N=1887) [32]. These comparisons included rash, neutropenias, and elevated LFTs. In the BAN Study, possible NVP hypersensitivity was noted in ~ 2% of infants receiving prophylaxis [44]. Additional information can be found in the Viramune package insert.

NVP has been shown to be effective for infant prophylaxis in the studies mentioned above, is recommended prophylaxis by the WHO, and because it has less potential to cause severe anemias when used in conjunction with CTX, we believe it is preferable to ZDV in this study setting (see discussion in Section 10.X below).

4. STUDY DESIGN AND STUDY SITES

4.1 General study design. This study will be a factorial design, double-blinded randomized trial comparing 1) CTX versus placebo (started from 14-34 days and continued to 15 months) to evaluate the benefit of CTX to improve infant survival and 2) a randomized BF intervention of 6 vs. 12 months of BF to evaluate the benefit of longer BF for HIV-free survival among HIV-exposed infants.

Infants born to HIV-infected women who choose to BF will be eligible for both randomizations. Infants born to HIV-infected women who choose to FF from delivery or who are no longer BF at the time of randomization to CTX/placebo will be eligible only for the CTX/placebo intervention. All HIV-exposed infants will be eligible for the CTX/placebo intervention, regardless of feeding method. Counseling regarding the following approaches to postnatal PMTCT for HIV-infected women will occur antenatally or at delivery in order to minimize postnatal MTCT for BF infants: 1) exclusive breastfeeding with infant NVP antiretroviral prophylaxis, among mothers who are not receiving HAART, or who have received less than 6 weeks of HAART, and 2) exclusive BF among mothers who have been receiving HAART for at least 6 weeks.

4.2 Study sites. This project will be carried out at the following established BHP clinical research sites in Botswana. This list may also be expanded to further include additional clinical research sites.

Molepolole. The study clinic will be located at Scottish Livingstone Hospital. Participating health clinics located within Molepolole (primarily Kgosing, Bokaa, MCC, Borakalalo, Boribamo, Scottish Livingstone, Phuthadikobo) will be involved in recruitment. Study enrollment and post-partum follow-up for participants from Molepolole will be performed at the study clinic located at Scottish Livingstone Hospital. Additional recruiting clinics may be added as necessary as the study accrual proceeds.

Gaborone. The study clinic will be located at Princess Marina Hospital. Health clinics located within Gaborone providing antenatal care will be involved in recruitment. Study enrollment and post-partum follow-up for participants from Gaborone will be performed at the study clinic located at Princess Marina Hospital. Additional recruiting clinics may be added as necessary as the study accrual proceeds.

Lobatse. The study clinic will be located at Athlone Hospital. Participating health clinics located within Lobatse (including Woodhall, Peleng East, Peleng Central, Tsopeng, Athlone) will be involved in recruitment. Study enrollment and post-partum follow-up for participants from Lobatse will be performed at the study clinic located at Athlone Hospital. Additional recruiting clinics may be added as necessary as the study accrual proceeds.

(if required) Mochudi. The study clinic will be located at Deborah Retief Memorial (DRM) Hospital. Participating health clinics located within Mochudi (including Clinic I, Clinic II, Makakatlela, Phaphane, Boseja, Deborah Retief Memorial) will be involved in recruitment. Study enrollment and post-partum follow-up for participants from Mochudi will be performed at the study clinic located at DRM Hospital. Additional recruiting clinics in Mochudi may be added as necessary as the study accrual proceeds.

Laboratory facilities. HIV ELISA, hematology, chemistries, Western blot confirmation of indeterminate samples, infant diagnostic PCR tests, CD4+ cell counts, HIV-1 RNA

measurements, breast milk processing, and plasma/cell separation will be carried out at the Botswana-Harvard HIV Reference Laboratory, adjacent to Princess Marina Hospital in Gaborone. Other assays (such as micronutrient or immunologic assays) will be carried out in Botswana, at the Botswana-Harvard HIV reference or other lab, whenever possible; this may entail bringing experts and expertise to Botswana to train local scientists to conduct new assays, for purposes of capacity building, whenever this is feasible (when local testing is simply not feasible due to issues of complexity or cost, this testing may be performed in specialty labs outside of Botswana). All laboratory samples will be transported daily from the sites appropriately using standard operating procedures.

5. SELECTION AND ENROLLMENT OF PARTICIPANTS

5.1. Inclusion/Exclusion Criteria

5.1.1 Inclusion/Exclusion Criteria for Interventional component of the study among HIV-infected women and their HIV-exposed infants

Inclusion/exclusion criteria for **study entry**

HIV-infected pregnant and postpartum women will be eligible to enroll for the interventional component of this study. The following inclusion and exclusion criteria will be used for women/infants participating in the randomization/interventional trial:

Inclusion criteria:

- HIV-infected women: either written health record documentation of a prior positive HIV test (ELISA, dual rapid test, or detectable HIV-1 RNA); or study-conducted positive HIV test (ELISA, dual rapid test, or detectable HIV-1 RNA)
- $\geq$ 26 weeks gestation and $\leq$ 34 days postpartum.
- Women must be $\geq$ 18 years of age and willing/able to sign informed consent.
- Women and infants must be able to follow up regularly at a study clinic through 18 months postpartum.
- Botswana citizen
- Not currently incarcerated

Exclusion criteria:

Antepartum:

- Known infant anomalies resulting in a high probability that the infant will not survive to 18 months.

Postpartum:

- Known HIV-infected infant (documented positive HIV PCR prior to randomization), or infant medical condition making survival to 18 months unlikely.
- Mothers with enrollment CD4$<$250 cells/mm^3 who choose to BF but who do not wish to start HAART (or who do not wish to continue HAART through at least the period of breastfeeding) cannot enroll in the trial

Inclusion/exclusion criteria for **randomization:**

Inclusion criteria for feeding randomization:

• Women must be willing to breastfeed for up to 12 months, and to stop at 6 months, depending upon their feeding assignment.

• Note: women who choose to BF but who do not wish to participate in the feeding randomization may still take part in the study and undergo randomization to CTX/placebo

• Note: women with twins who participate in the BF randomization will undergo a linked randomization whereby both infants will be randomized to the same BF duration arm.

Exclusion criteria for infant randomization:

All enrolled infants should be randomized, with the following exceptions:

1) Infants with a positive HIV PCR result (unless the test is confirmed to be a false positive)

2) Infants with known allergy to sulfa drugs (including but not limited to CTX)

3) Infants with illness/medical condition making survival to 18 months unlikely

4) Infants with clinical jaundice, or with known and documented current grade 4 anemia or grade 4 neutropenia (or *symptomatic* grade 3 anemia/neutropenia) should not be randomized. All such infants should be re-evaluated at least weekly during the randomization window to determine whether randomization has become possible (i.e., if jaundice has resolved, or if other events have resolved to grade 2 or lower).

5) Infants with other illness/medical condition that makes initiation of study CTX/placebo during the randomization window contraindicated; these instances should be discussed on a case by cases with the protocol team.

6) Infants who were born at < 36 weeks gestational age, **or** who weigh < 2.5 kg at 14 days, should not be randomized until they are 28-34 days of age

Infants who were enrolled in the study but who cannot be randomized should be taken off-study

5.2. Recruitment and accrual. Screening and enrollment for this study will occur at antenatal care (ANC) clinics associated with 3-4 district hospitals in southern Botswana, which may include: Princess Marina Hospital (Gaborone), Scottish Livingstone Hospital (Molepolole), Athlone Hospital (Lobatse), and Deborah Retief Hospital (Mochudi) or in the maternity wards at any of these four hospitals. There are approximately 9000 births per year combined between these 4 district hospitals. An advantage to performing this study in Botswana is that the vast majority of pregnant women receive antenatal care, and are accessible for enrollment during pregnancy. In recent surveillance, 99% of Botswana citizens who delivered at Princess Marina Hospital in Gaborone had registered at an antenatal clinic during pregnancy (BHP, unpublished data, 2009).

Although infant randomization will occur from 14-34 days, and maternal enrollment will be allowed up to 34 days postpartum, a portion of the recruiting effort will occur in the antenatal period for several reasons: 1) the antenatal period is an ideal time to access women who are receiving care at government ANC clinics, and it will allow women time to be adequately

counseled and consented; 2) antenatal enrollment allows for women to be counseled about feeding options and to make an appropriate choice of feeding method from birth; 3) antenatal enrollment will allow for a better assessment of mortality in the first 4 weeks of life which will improve our overall assessment of infant mortality and the potential impact of the randomized objectives; and 4) pre-randomization losses should have no impact on the validity of the randomized trial and have been accounted for in the overall accrual estimates.

Our experience recruiting at these same sites for the Mashi and Mma Bana Studies allows us to estimate accrual with precision. In these previous studies, we enrolled ~ 40 participants per month but we were limited by large losses of potential participants who were never referred to our study sites to be enrolled before 34 weeks gestation. In the current study, women can be enrolled from 26 weeks gestation to 34 days postpartum (most are expected to enroll either antenatally or within 72 hrs postpartum), and we will have direct access to ALL HIV+ women seen at each referral clinic and at the maternity wards. HAART receipt and feeding choice are not reasons for ineligibility, so virtually all HIV-infected women will be eligible for the study. In the Mma Bana Study, the single greatest source of "loss" to accrual was that of 4,195 HIV+ women identified in government screening at the 4 sites combined, only 30% were referred to our study sites to hear about the study and potentially enroll by 34 weeks gestation (BHP, unpublished data, 2008). By accessing the additional 70% of HIV+ women that we were missing in Mashi and Mma Bana accrual (primarily because of referral too late in gestation), we can markedly improve accrual. We do not anticipate that many women will decline to be in the study. For HIV-infected women who choose to BF, the randomization is for a recommended duration, and therefore a woman's actual feeding plans do not need to disqualify her from this aspect of the study (or the CTX/placebo intervention), and we expect good study acceptance. In the Mma Bana Study, ~9% cited the desire to FF rather than BF as a reason for declining the study, and this will no longer be a concern.

We expect < 5% of women who choose to BF to do so for *longer* than the recommended weaning time at either 6 or 12 months, and thus almost all women will have the high-risk weaning period covered by either CTX or placebo which will extend to 15 months. Overall, the greater numbers available by the ability of recruiters to work directly at referral clinics; to enroll later in pregnancy and at multiple antenatal visits and in the postpartum period in the maternity ward; and to enroll women no matter what their feeding intentions will allow for a doubling of our past accrual (even with the possible reduction of sites from 4 to 3). Thus, we estimate that ~ 80 women per month will be enrolled at 3 sites.

We plan to stratify the randomization to ensure that there are an equal number of CTX vs. placebo comparisons within each of the main feeding groups (BF for 6 months, BF for 12 months, and FF from birth). In addition, we plan to stratify the BF randomization such that each method of intended MTCT prophylaxis (maternal HAART and infant NVP) is equally distributed between 6 vs. 12 month randomization arms. This strategy will allow for balanced comparisons and will avoid potential bias by feeding method (for CTX intervention) or by prophylaxis method (for BF intervention) if unexpected differences in the magnitude or direction of effect are present between these factors.

5.3. Informed consent and participant reimbursement.

Mothers will undergo an informed consent process (in the language that the volunteer is most comfortable speaking—generally in Setswana) by trained study staff. Volunteers will sign (or mark) a written informed consent/permission for their and their child's participation in this study, respectively. Children who are under the care of a guardian may be enrolled with the consent/permission of the guardian. We will offer reimbursement for participant transport, approximately 30-50 pula per attended scheduled visit, to compensate for participant time commitment (rate to be approved by the Botswana HRDC).

5.4 Randomization Procedure

For HIV-infected women BF and agreeing to be randomized to 6 vs 12 months of BF, factorial randomization will be as permuted blocks by study site into one of 4 possible groups – CTX/6 mos BF, CTX/12 mos BF, placebo/6mos BF, placebo/12mos BF. Within these 4 groups, the method of MTCT prophylaxis will be balanced to keep similar numbers of "maternal HAART" prophylaxis in all 4 groups and similar number of "infant NVP" prophylaxis in all 4 groups. Formula feeding HIV-exposed uninfected infants (or BF mothers/infants who decline randomization to 6 vs. 12 months of BF) will be randomized to CTX vs. placebo only, also using permuted blocks. Randomization is also stratified by study site. The electronic data system (EDS) will assign randomization groups according to this protocol, upon request by sites for a randomization (and upon confirmation of eligibility). Multiple births (twins, triplets etc) will be randomized to the same arm (CTX or placebo) as one another (and among mothers choosing to breastfeed, to the same duration of breastfeeding).

<u>Timing of Randomization (also, see Section 6.2):</u>

The randomization window is 14-34 days of life, inclusive (with the date of birth = day 1). Infants who were born at ≥ 36 weeks gestation and who weigh ≥ 2.5 kg should be randomized as close to 14 days of age as possible, to maximize the potential benefit of CTX/placebo (but can be randomized at any time during the 14-34 day window). Infants who were born at < 36 weeks gestational age, **or** who weigh < 2.5 kg at 14 days, should not be randomized until they are 28-34 days of age (at which point they can be randomized regardless of gestational age or weight, as in Version 1.0 of the protocol).

<u>Special Circumstances for Management of Anemia or Neutropenia prior to / at randomization:</u>

1. Grade 4 anemia or neutropenia that is first noted from a clinically-driven laboratory prior to randomization (result available before randomization):

> Randomization is deferred until < Grade 4 (or < Grade 3 if symptoms); no randomization without documented reduction to < Grade 4 (or < Grade 3 if symptoms) within randomization window**.**
>
> Asymptomatic Grade 3 anemia or neutropenia will be followed carefully, but does not require delay in initiation of CTX/placebo unless the infant becomes symptomatic, or unless the Grade 3 abnormality has persisted for 4 weeks or longer without improvement.

2. Grade 4 anemia or neutropenia that is first noted from the day-of-randomization laboratory (result NOT available before randomization):

If the Grade 4 anemia or neutropenia is confirmed with a 2nd blood sample, the CTX will be held until anemia or neutropenia resolves to Grade 2 or lower (if symptoms) or to Grade 3 or lower (if no symptoms), at which point the CTX will be restarted.

3. Grade 3 anemia or neutropenia that is first noted from the day-of-randomization laboratory (result NOT available before randomization):

Managed as for Grade 3 anemia or neutropenia above.

6.0. STUDY INTERVENTIONS AND EVALUATIONS

6.1 Study medication dosing and breastfeeding intervention, HIV-exposed infants

6.1.1 Infant CTX/placebo

- Randomization (from 14 to 34 days of life) to 6 months of age: CTX/placebo 100mg/20mg once daily, or 2.5 mL once daily of syrup from a 200mg/40mg per 5mL suspension
    - Infants who were born at < 36 weeks gestational age, **or** who weigh < 2.5 kg at 14 days, should not start study CTX/placebo (should not be randomized) until they are 28-34 days of age
    - Infants who were born at ≥ 36 weeks gestation and who weigh ≥ 2.5 kg should start study CTX/placebo (should be randomized) as close to 14 days of age as possible, to maximize the potential benefit of CTX/placebo (but can start CTX/placebo at any time during the 14-34 day window)

- 6 months to 15 months of age: CTX/placebo dose increases to 200mg/40mg once daily, or 5 mL once daily of either of syrup from a 200mg/40mg per 5mL suspension

Note: infant CTX/placebo should be started on the day of randomization (preferably, at the study clinic) whenever possible.

6.1.2 Infant NVP

- Birth to 4 weeks of age is 10 mg NVP once daily if infant weight is 2000-2500 gm at birth, and 15 mg NVP daily if >2500 gm at birth
- 4 weeks to 6 months of age is 20 mg NVP once daily
- 6 months to 9 months of age: 30 mg NVP once daily
- 9 months to 12 months of age: 40 mg NVP once daily

For infants weighing < 2000 gm at birth, the following NVP dosing should be used:

- >/=1800 gm - < 2000 gm at birth: 5 mg NVP once daily 0-14 days of age, 10 mg NVP once daily 15-42 days of age, standard dosing thereafter (20 mg NVP once daily to 6 months)
- < 1800 gm at birth: 2 mg/kg NVP once daily 0-14 days of life, 10 mg NVP once daily 15-42 days of age, standard dosing thereafter (20 mg NVP once daily to 6 months)

Note: If NVP is contra-indicated or not tolerated in the first month of life, ZDV (per government guidelines) may be substituted until 28 days of life, if clinically appropriate. Infants receiving ZDV rather than NVP should be randomized to CTX/placebo after discontinuation of ZDV.

6.1.3 Feeding/weaning counseling

- Mothers choosing to breastfeed: the weaning and infant prophylaxis plan should be discussed at the randomization visit (and then thereafter as needed). The mother is counseled that weaning should occur over a 4-week period, when it occurs. The study clinician will write for infant formula in the infant's "Under 5 Health Booklet", if early weaning anticipated before the next visit.
- Mothers choosing to formula feed: counseling will be provided on safe feeding choices and practices, including safe use and preparation of formula, both at enrollment and throughout the study as needed.

6.2 Schedule of evaluations, <u>HIV-infected mothers and their infants</u>

<u>Antenatal Period: Maternal Enrollment</u>

- Information about the study will be made available at antenatal visits to sensitize women to the study
- <u>>/= 26weeks of pregnancy, but prior to onset of labor:</u> Consent and enrollment may occur if 1) Positive HIV status is established by documented HIV status in medical records, and 2) other eligibility criteria are met
- Record demographics, pregnancy and HIV / antiretroviral history
- Discuss and provide counseling related to feeding options
- For consented and enrolled participants, mark obstetric cards with study code or BID and maternal feeding choice (once decision made)

<u>Maternal "Delivery" Visit (up to 34 days postpartum)</u>

- If not enrolled: Enrollment may occur any time up to 34 days postpartum if 1) Positive HIV status is established / documented, and 2) other eligibility criteria are met. If the mother is enrolled at or after delivery, complete the evaluations and steps summarized in section above regarding Maternal Enrollment.
- Maternal delivery CRF
- Maternal CD4, HIV-1 RNA, CBC
  (*Note: Confirmatory HIV ELISA should be performed if maternal HIV RNA returns undetectable in a woman not receiving ARVs)*

<u>Infant "Birth Visit" (up to 34 days of life)</u>

- Confirm feeding method and ARV receipt.
- HIV-exposed uninfected infants will receive at least 4 weeks NVP prophylaxis,regardless of feeding choice (ZDV prophylaxis may be substituted if NVP is contraindicated) (see Section 6.1.2 for dosing)
- Discuss safe formula use, if mother chose FF

- Infant HIV DNA PCR (by dried blood spot or venous blood)
  (Note: if HIV DNA PCR is positive, infant should be recalled for confirmatory PCR;, infant NVP is stopped if confirmatory PCR is positive and infant is referred for HAART; and infants with confirmed positive HIV DNA PCR should stop study CTX/placebo and start open-label CTX at 4-6 weeks of age, per government protocol)

<u>Randomization Visit: (from 14 to 34 days of life, inclusive; enrollment and randomization can occur on the same day)</u>

- The following infants should **not** be randomized: infants with a positive HIV PCR result (unless the test is confirmed to be a false positive), or infants with clinical jaundice, with known and documented current grade 4 anemia or neutropenia (or *symptomatic* grade 3 anemia/neutropenia), or with known allergy to sulfa/CTX. These infants should be should be taken off-study if randomization is not possible by 34 days of age. See sections 5.1.1 and 5.4 for details)
- Infants who were born at $\geq$ 36 weeks gestation and who weigh $\geq$ 2.5 kg should be randomized as close to 14 days of age as possible, to maximize the potential benefit of CTX/placebo (but can be randomized at any time during the 14-34 day window)
- Infants who were born at < 36 weeks gestational age, **or** who weigh < 2.5 kg at 14 days, should not be randomized until they are 28-34 days of age (at which point they can be randomized regardless of gestational age or weight, as in Version 1.0 of the protocol). Likewise, infants receiving ZDV rather than NVP as MTCT prophylaxis (e.g. due to NVP intolerance) should be randomized after ZDV is discontinued at 28 days.
  Dosing of randomized CTX/placebo is described in Section 6.1.1
- Infants of women choosing to breastfeed are simultaneously randomized to continue breastfeeding until either 6 months or 12 months of age
- Infants of women choosing to formula feed are randomized only to CTX/placebo
- Counseling should be provided regarding weaning (for women choosing to BF) and safe formula use (for women choosing to formula feed; see Section 6.1.3)
- The importance of continuing infant NVP prophylaxis through the one month visit should be reinforced with all HIV-infected women.
- FF infants should discontinue NVP at 4 weeks of age.
- Mothers who opt to BF but who are not taking HAART should be reminded that infant NVP will be continued until 1 week after BF cessation.
- BF infants of mothers who have received at least 6 weeks of HAART by 4 weeks of age and whose mothers are continuing HAART should discontinue infant NVP at 4 weeks.
- BF infants whose mothers have received less than 6 weeks of HAART (and who are continuing HAART) should continue NVP prophylaxis until the mother has received 6 weeks of continuous HAART (at which point infant NVP prophylaxis may be stopped).
- Among women on HAART, the importance of adherence and side effect profile of study medications should be reviewed
- Women eligible for HAART by enrollment CD4 cell count or AIDS-defining illness will be referred to government clinics for start of HAART, but remain on study
- Share maternal HIV-1 RNA results and infant birth PCR results with the participant
- Perform infant clinical assessment / physical examination, and maternal clinical assessment

- Infant HIV PCR testing by dried blood spot or venous blood *(Note: If this day-of-randomization HIV DNA PCR is positive, infants will be recalled, started or maintained on CTX, and referred for HAART. These infants will not be included in the primary study analyses. However, they will continue to be followed in the study in Version 2.0, and may contribute to clinical outcomes. No labs will be drawn on these infants.)*
- Obtain infant CBC, stored plasma/cells *(Note: This CBC is drawn on the day of randomization, so result not available that day. If Hgb = Grade 4 from this draw, or from another draw after randomization, infants will be recalled and CTX held until < Grade 4. However, this infant has been randomized and remains on study, and study drug is managed as per SOPs for anemia)*
- Maternal stored plasma/cells and breast milk (the latter only in BF mothers)
- Review methods of contacting study staff if concerning infant side effects are noted

<u>Follow-up Visits at Infant Ages 2, 3, 6, 9, 12, 15, 18 Months</u>

- Review infant CTX and NVP dosing and adherence (and maternal HAART status and eligibility), as applicable
- Feeding counseling, discuss weaning plan (if not weaned), feeding reporting, routine care, event reporting, labs per schedule
- For HIV-infected women opting to BF, encourage adherence to randomized feeding arm and confirm weaning per protocol.
- Clinical assessment and physical examination of infant; clinical assessment of mother
- Infant HIV PCR testing for all HIV-exposed infants who breastfed for any period since the previous visit, at months 3, 6, 9, 12, and 15 months, or for any infant going off-study before 18 months of age. *(Note: If PCR positive, infants will be recalled for confirmatory DNA PCR; if the 2nd PCR is positive, the infant will be started on open-label CTX, and referred for HAART. If BF and receiving prophylactic NVP, this will be stopped upon confirmation by a second PCR)*
- ELISA at 18 months of age for all HIV-exposed infants
- Stored plasma/cells at 6 and 15 months of age
- Infant CBC at 3, 6, 15 and 18 months of age (and if clinically indicated, at other visits)
- Medication refills for infants taking study medications at designated scheduled visits and more frequently if needed between scheduled visits.
- CTX/placebo (and NVP, if infant still taking NVP) dose increased at 6 months
- Among BF infants still taking NVP (if mother not taking HAART), NVP dose increased at 9 months and NVP discontinued 1 week after BF cessation (12 months at latest)
- CTX/placebo stopped at 15 months

Table 1. Clinical and Laboratory Follow-up of HIV-Infected Mothers

MOTHERS	Ante-natal enroll-ment	Deliv	Randomization (14-34 days postpartum	2 months post partum	3 months post partum	6 months post partum	9 months post partum	12 months post partum	15 months post partum	18 months postpartum
CRF Questionnaires and clinical evaluations	X	X	X	X	X	X	X	X	X	X
Counseling	X	X	X	X	X	X	X	X	X	X
Hematology*		X	**							
CD4 – CD8 count		X [#]	**/ [#]							

Plasma Viral Load		X	**/***/%		***		***			
Stored Plasma, Cells		X	**							
Breast Milk& Storage			X							

* Hematology includes: hemoglobin (g/dl), Hematocrit (%), RBC (million/mm^3), MCV (microns), WBC (10^3/ cu mm), Platelets (10^3/cu mm).
\# If on HAART, identify and record pre-HAART CD4 cell count if available
** Re-draw as baseline values if "delivery visit" > 31 days from 1 month visit, or if missing delivery labs.
*** Only recheck HIV-1 RNA among BF women with identified HAART adherence concerns (or if previously detectable > 400 copies/mL) while on HAART, to evaluate safety of ongoing BF
& If breastfeeding
% HIV-1 RNA testing will not be run in real-time unless required for clinical purposes

Table 2. Clinical and Laboratory Follow-up of HIV-Exposed Infants£

INFANTS	Birth	Rando (14-34 days of age)	2 months	3 months	6 months	9 months	12 months	15 months	18 months ∝
CRF Questionnaire and clinical evaluations	X	X	X	X	X	X	X	X	X
Physical exam	X	X	X	X	X	X	X	X	X
Hematology*		X		X	X			X	X%
Stored plasma/cells**		X			X			X	
HIV DNA PCR	X	X		X&	X&	X&	X&	X&	
HIV ELISA									X

£ HIV+ infants who remain on study after randomization do not have study labs drawn

* Hematology includes: hemoglobin (g/dl), Hematocrit (%), RBC (million/mm^3), MCV (microns), WBC (10^3/ cu mm), Platelets (10^3/cu mm).
** Separate sample; stored for micronutrient/immunologic testing
∝ "Off study" forms and laboratories will be performed prior to study discontinuation, whether at 18 months postpartum or at an earlier date
.& For infants BF since last PCR test (run in real-time, not stored)
% If Grade 2 or higher at 15 months.

6.4 Timing of evaluations

Screening evaluations and enrollment should occur from 26 weeks gestation to 34 days postpartum (most are expected in the antenatal or immediate postpartum period).

Infant randomization must occur ≥ 14 days of life and ≤ 34 days of life. Enrolled participants who fail to randomize will be considered as "pre-randomization loss to follow-up" and will not have further study visits if they present later.

Post-randomization scheduled evaluations should occur after enrollment and at the weeks/months indicated, within the following windows: +/- 14 days around the 2- and 3-month visits; and +/- 45 days around the 6, 9, 12, 15 and 18-month visits. Every attempt should be made to conduct the visit as close as possible to the target visit date, and barring that, within the target visit window. However, if a visit window is missed, an attempt should still be made to conduct the target visit (and to perform laboratories required at the missed visit) outside of the specified visit window.

Missed visits
If a scheduled visit is missed and the next visit at the clinic falls between two scheduled visits, the next visit should include major monitoring parameters from the previous missed visit. If a

visit is missed, the schedule of visits should not be "reset" but should remain as if the visit were not missed.

Ill visits

Unscheduled ill visits may be seen at the discretion of study staff. If the ill visit should fall within the allowable visit window of the next regularly scheduled study visit and the participant indicates that she/the baby will not be able to attend the next scheduled visit, the ill-visit can substitute for the scheduled visit at the discretion of the study physician. In such a case, all information/tests scheduled for the routine visit should be obtained and reported to the Data Centre.

When an ill visit is not considered a scheduled visit, clinical events that meet reporting criteria for the study should be noted in the patient medical record (non-source document) and also recorded as notes in a clinical flow sheet in the patient's CRF folder. The complete event will then be reported on case report forms (CRFs) and submitted to the Data Centre *at the next routine scheduled visit*. The participant should be reminded to attend the next regularly scheduled study visit. However, should the ill visit constitute a serious adverse event that requires EAE reporting, the EAE form should be completed and submitted to the Data Centre within the specified time frame.

6.5 Laboratory testing panels

Hematology

Hemoglobin, hematocrit, red blood cells (RBC), mean corpuscular volume (MCV), white blood cell count (WBC), differential WBC, absolute neutrophil count (ANC), and platelets.

Blood Chemistries (if required)

Sodium, potassium, chloride, glucose, bicarbonate, BUN, creatinine, ALT, AST, and bilirubin.

CD4+/CD8+

Determinations of CD4+ and CD8+ cell counts and subset percentage evaluations should be performed at the approved BHP laboratory in Gaborone, throughout the course of the study. All CD4+ and CD8+ cell count results will be recorded on the CRFs.

Plasma HIV-1 RNA

All evaluations are to be done using the approved viral load assay, at the BHP study laboratory in Gaborone.

HIV DNA PCR

Whenever an HIV DNA PCR is required and it is the only sample required it will be acquired as a dried blood spot (DBS) on filter paper (See separate SOP). At visits at which blood is being collected by venipuncture, a sample for DNA PCR can either be collected in a tube or by filter paper DBS. For the birth sample, the DBS for HIV-1 DNA PCR should be obtained from a source other than cord blood (to avoid contamination) in a manner that will minimize number and volume of infant phlebotomy (e.g., from a heelstick). Qualitative HIV DNA PCR testing for infant HIV diagnosis will be performed at the BHP laboratory in Gaborone.

Stored Plasma
Stored plasma will be performed per laboratory SOP.

Stored cells
Cell pellets will be stored and frozen.

Stored breast milk
Whole milk, supernatant, and cell pellets will be stored at the protocol-specified time points among mothers who are breastfeeding.

Micronutrient/immunologic testing
Stored plasma, cells and breast milk will be used for testing for micronutrient levels (including Vitamin D), and immunologic testing (including humoral/antibody/cytokine level, and innate/cellular responses to pathogens or vaccines). This testing will occur for a subset of HIV+ women and their infants, and for HIV- women and their infants.

6.6 Clinical evaluations and reporting requirements

Mothers: maternal weight is recorded at each visit, and maternal height is recorded at enrollment. At all maternal visits (including enrollment), only a targeted physical examination is performed (in instances in which a clinical concern arises from participant history or laboratory results, targeted to assess these findings); maternal physical examination findings are not reported on case report forms. Maternal demographics, general health, obstetric, and HIV/ARV (if HIV-infected) history are recorded at enrollment. Maternal antiretrovirals during the current pregnancy are recorded, as are details related to labor and delivery. At subsequent visits, the following are evaluated and recorded on case report forms: all maternal antiretrovirals/antiretroviral changes, new Grade 3 or 4 diagnoses (or major chronic medical problems as summarized in case report forms), new WHO Stage III/IV illnesses, or hospitalizations. Mastitis is reported for breastfeeding women. Maternal signs/symptoms are not reported on case report forms (unless they constitute one of the reportable diagnoses, e.g. chronic diarrhea or weight loss that meet WHO illness reporting criteria). Maternal medications are not reported, other than antiretrovirals and maternal CTX prophylaxis.

Infants: infants undergo a full examination at birth (results are recorded); thereafter, only a targeted examination is performed if needed. Height, weight, and head circumference are recorded at birth and height and weight are recorded at every visit thereafter. Birth/delivery information is recorded on CRFs, including infant prophylactic antiretrovirals and vaccinations received, and congenital anomalies. All Grade 3 or 4 diagnoses will be recorded on CRF (as will WHO Stage III/IV HIV-related illnesses). In addition, all Grade 2-4 rash or hepatitis will be recorded on CRF. Vaccinations, hospitalizations, and relevant concomitant medications (from a targeted list included in the electronic data capture system) are recorded at each follow-up visit, as is feeding method. Infant adherence to and modifications in study CTX/placebo (and NVP, if relevant) are recorded at each visit (as are other antiretrovirals, if relevant -- e.g. HAART for HIV-infected infants). These are assessed with specific questions in the CRFs.

Note: all laboratory test results for tests run at the BHP lab (BHHRL) are automatically imported into the database.

6.7 Infant feeding.

We will ask mothers regarding their choice of likely infant feeding method antenatally and at delivery. At each postpartum visit, we will ask mothers/caregivers for information on actual infant feeding practice and document feeding by on CRFs. Counseling will occur re: appropriate infant feeding according to 1) IRB approved educational materials, or 2) Botswana feeding guidelines.

6.8 Medical care and referrals

Mothers and infants who qualify for HAART but are not yet receiving it should be referred to the appropriate local ARV clinic for treatment, with all of the relevant clinical and lab result documentation. HAART will not be provided through the study clinic (including treatment for hyperbilirubinemia, as needed). Participants will remain on study, and HAART receipt will be noted in appropriate CRFs. Mothers will also generally receive other medical care (including CTX prophylaxis, if they qualify) through their local clinics.

HIV-exposed breastfeeding infants will receive extended NVP prophylaxis through the study staff/pharmacist, if their HIV-infected mother is not already on HAART for at least six weeks.

Most of the antenatal, well-baby, and general medical care of mothers and infants should be provided through the participants' usual health clinics. However, if a participant (maternal or baby) visits the study clinic with a complaint or illness, then the study staff will assist her/him with immediate care and referral as needed.

6.9 Data Management. Data will be entered by clinical study staff onto standardized case report forms (either paper or electronic) at the sites. After quality assurance checks, data will be entered by data clerks from the case report forms or direct electronic transfer will occur into the BHP web-based database. A data manager at the BHP Data Management Centre will be assigned to this study, and will incorporate basic error checking capability to minimize data entry errors and to allow rapid querying of the sites for illogical or missing data. The data manager will generate monthly accrual reports (by site and cohort), as well as periodic reports (approximately 3-monthly) that will summarize basic demographic data and reported aggregate diagnoses, to allow real-time review of enrollment/data.

7.0 LOSS TO FOLLOW-UP, DEATH, OFF-STUDY

7.1 Participants Lost to Follow-up

Collecting any intention to move or change of address during each visit will minimize the loss of contact with participants. In case of absence at a scheduled visit, a study nurse or counselor will attempt to locate the mother/child by phone or home visit (if permission to do so had been granted by the participant). Efforts will be made to keep all participants in study follow-up. Specifically, women who indicate that they will be unable to return at scheduled intervals for study medications and/or evaluation, will be asked about their willingness to remain on study, with their infant off study medication for the purposes of assessing infant HIV-free survival

status. However, loss to follow-up may occur after at least 3 consecutively missed visits with no contact despite repeated attempts.
Note: If a participant who was determined to be lost to follow-up subsequently contacts the study site, the Data Centre should be notified and follow-up on the appropriate study should be re-established in consultation with the Data Centre.

7.2 Maternal or infant death

In the case of mother or infant death after enrollment (including stillbirth, if a mother has enrolled during pregnancy), a Death Form should be completed within 3 days of becoming aware of the death. In the case of an infant death occurring within 30 days after discontinuation of the study medication, where the death is both unexpected and related or possibly related to the study medication, an EAE form must be completed and submitted to the DMC, the study sponsor and study IRBs within 3 working days of the site becoming aware of the event. If a woman or child dies in a health facility, events prior to and at the time of death will be collected from hospital records and a verbal autopsy CRF will be completed. If death occurs outside the hospital setting, a verbal autopsy CRF will be completed upon interview of the mother or guardian, in the event of an infant's death, or a relative in the event of a maternal death and the Death Form will be completed. If the mother of an infant dies, infant follow-up will continue throughout the study period. The purpose of the study will be explained to the guardian of the child. We will ask the mother, where possible, to identify an individual to whom she would like the study team to share information about her baby's health in the event of her absence. This information will be documented on the locator form. Additional support will be sought from social services.

7.3 Off Study

- Request by the participant to withdraw
- Participant misses 3 consecutive study visits (despite attempts to locate and discuss with participant) may be a criterion for being taken off-study
- Participant judged by the investigator to be at significant risk of failing to comply with the provisions of the protocol as to cause harm to self or seriously interfere with the validity of the study results.
- The study may be discontinued by NICHD, the IRB/ECs, Office of Human Research Protection (OHRP), Botswana Government or other government agencies as part of their duties to ensure that research participants are protected.

8.0 ADVERSE EVENT REPORTING AND TOXICITY MANAGEMENT

8.1 Expedited Adverse Event Reporting

Real-time adverse event reporting (within 3 business days of recognition) to the study sponsor (NICHD) and study IRBs will occur for any *infant* serious adverse events (i) that are **both unexpected and related/possibly related to research**; (ii) that occur while the infant is enrolled in the study or that occur within 30 days of the conclusion of the infant's participation in the study, of which the investigator becomes aware.

In addition, real-time EAE reporting will also occur for:

a) All infant deaths that are possibly (or probably or definitely) related to study drug, regardless of expectedness [note: infant deaths that are *definitely not* related to study CTX/placebo or extended infant NVP prophylaxis do not need to be reported as EAEs]; and
b) All incidents of Stevens Johnsons Syndrome experienced by an infant after a study drug (extended NVP prophylaxis, and/or CTX/placebo) has been started
Both study-provided CTX/placebo and NVP in infants are considered study drug.

This EAE reporting is only applicable to infants, as mothers will not receive any study drugs.

8.2 Quarterly Adverse Event Reporting

Information is collected on SAEs. A serious adverse event is defined as any event (expected or unexpected, and regardless of relationship to study drug) temporally associated with the subject's participation in research that meets any of the following criteria:

- Results in death
- Is life threatening
- Requires hospitalization/prolongation of hospitalization
- Results in congenital anomaly
- Results in persistent or significant disability/incapacity
- Required intervention to prevent permanent impairment/damage

As described in Section 8.1, only the serious adverse events that also meet criteria for unexpectedness and relatedness to study drug will be reported as EASs within 3 business days of the site becoming aware to the sponsor and study IRBs, with the exceptions noted above.

However, the following infant events that occur after infant enrollment will be included in quarterly AE reports to NICHD and annual reports to the IRB, as well as in DSMB reports:

a. Grade 3/4 clinical events
b. Deaths
c. Steven's Johnson Syndrome events
d. Hospitalizations
e. Persistent disability/incapacities
f. Grade 3/4 hemoglobin
g. Grade 3/4 neutropenia
h. Grade 3/4 platelets

Note: congenital anomalies/stillbirths will not be included in aggregate AE reports, as mothers do not receive study product.

8.3 Toxicity Management

The most current version of the Division of AIDS (DAIDS) standardized Toxicity Table for Grading Severity of Adult and Pediatric Adverse Experiences should be used (at the time of V2.0

of this protocol, the December 2004 version is applicable) to screen for eligibility and to report adverse events for pregnant and postpartum women, and among infants (toxicity table is available at http://rcc.tech-res-intl.com.)

This Toxicity Management section refers to management of toxicities that occur among infants who are taking study product (CTX/placebo and/or extended NVP prophylaxis). Please also refer to specific SOPs for management of toxicities.

For breastfeeding infants stopping NVP prophylaxis: In all instances where toxicity management requires discontinuation of NVP prophylaxis in a BF infant, and where the mother has not been receiving HAART for at least 6 weeks, infants should immediately and completely stop breastfeeding and their mothers should be provided with formula and counseling regarding its preparation. Mothers should be instructed to express and discard breast milk to prevent discomfort. If the infant NVP cessation is expected to be temporary, mothers may continue this practice until NVP resumes; otherwise, mothers should gradually reduce expression and stop over a period of days and should not resume breastfeeding in the future for this infant.

8.3.1. Dosage Modification Instructions

No study drugs will have dosages reduced because of toxicity.

8.3.2 Management for Rashes and Hypersensitivity Reactions

All rashes are graded based on the DAIDS toxicity table (December 2004).

Grade 1 or 2 rashes

Study products may be continued with close observation, at the discretion of the study clinician. If an isolated Grade 1 or 2 rash does not resolve within 14 days of onset, further management should be discussed with the study team. Other potential causes of rash should be investigated and treated, and potential causative agents (such as lotions/creams/soaps) should be discontinued.

If there is a definitive alternative diagnosis for the rash other than study medication (for example, infant acne, diaper rash, varicella), then ALT and FBC do not need to be measured. However, if there is no definitive explanation for the rash/skin reaction, the infant must have ALT and FBC with differential drawn and value reviewed.

If a Grade 2 or higher ALT (and/or eosinophilia) are present in combination with a Grade 1 or 2 rash without other explanation, then study CTX/placebo should be held and NVP should be stopped (and the mother counseled to wean immediately and provided with formula). Re-initiation of study CTX/placebo should be discussed with the protocol team.

Grade 3 rash with no definitive alternative explanation (with no mucosal involvement)

Infant CTX/placebo should be held and infant NVP prophylaxis should be permanently discontinued (and breastfeeding babies weaned immediately to formula). In addition, any products or non-essential medicines that could be causing the rash should be discontinued. ALT and FBC with differential should be drawn, and the baby should be evaluated clinically approximately weekly, until the rash resolves to Grade 1 or lower. If the Grade 3 rash was very

likely due to another diagnosis or drug, then the CTX/placebo may be restarted with close follow-up once the rash and ALT are Grade 1 or lower, in consultation of the protocol team.

Grade 4 rash (or Grade 3 rash with mucosal involvement, or suspected hypersensitivity)
Immediately and permanently discontinue infant CTX/placebo and NVP prophylaxis and refer to the hospital for further clinical care/management.

If infant NVP prophylaxis is stopped before 4 weeks of age, then infant ZDV may be substituted until 28 days of age. If NVP is permanently discontinued, the mother should also be advised to wean from BF to FF, unless she is receiving HAART and has been on HAART for 6 weeks (or has documented viral suppression; HIV RNA may be drawn for this purpose and run "stat" during which time the mother should be counseled to express breast milk and discard it while formula feeding the infant).

8.3.3 Guidelines for Managing Anemia or Neutropenia

Given the lack of significant hematologic toxicity associated with infant NVP prophylaxis in the controlled clinical trials to date, the following section pertains to the management of study CTX/placebo.

Grade 1 or 2 Anemia or Neutropenia
Study drug may be continued.

Grade 3 Anemia or Neutropenia
If the anemia/neutropenia can be attributed to a specific cause other than study CTX/placebo, then study CTX/placebo may be continued with careful monitoring after discussion with the protocol team (and the suspected specific cause addressed).

If the participant is *symptomatic* in the face of confirmed Grade 3 anemia/neutropenia, and the anemia/neutropenia cannot be attributed to a specific cause, then any medications potentially contributing to the anemia/neutropenia (e.g. infant AZT, maternal AZT if the baby is breastfeeding, and study CTX/placebo) should be held or substituted.

If the participant is *not symptomatic* in the face of confirmed Grade 3 anemia/neutropenia, then study CTX/placebo can be continued with careful clinical and hematologic monitoring for up to 2 weeks, in consultation with the study team. If an anemia persists at Grade 3 for 2 weeks or more; or if a neutropenia persists at Grade 3 for 4 weeks or more (or if either worsens to Grade 4 or becomes symptomatic), then medications potentially contributing to the anemia/neutropenia (including study CTX/placebo) should be held.

Participants should be monitored closely, with frequent (every 1-2 weeks) repeat haemoglobin testing (or a post-transfusion value to document resolution if transfusion is performed) until the anemia reaches ≤ Grade 2. Participants should also be monitored (by phone or visit) for signs/symptoms of infection, in cases of Grade 3 neutropenia.

Once the anemia/neutropenia reaches Grade 2 or lower, study CTX/placebo can be restarted with careful follow-up. If confirmed Grade 3 or worse anemia/neutropenia recurs, then the same procedures noted for the first incident should be followed. If study drug needs to be discontinued a second time, it should not be restarted a third time after resolution.

Grade 4 Anemia or Neutropenia
In general, the following measures will not be undertaken until the Grade 4 value is confirmed. However, if there is serious clinical concern (based upon participant's signs/symptoms) for symptomatic and severe anemia/neutropenia, then these measures do not need to await confirmation of the Grade 4 value.

Management of the confirmed Grade 4 anemia or neutropenia will be as follows:

Immediately discontinue or substitute any medications potentially contributing to the anemia/neutropenia (including AZT, CTX/placebo), and do not replace with other medications that might contribute to anemia/neutropenia. Continue to monitor participant frequently and referred for hospitalization if symptomatic anemia. Unblinding may occur. This decision will only occur in consultation with the study team, and referring to the SOP for unblinding (and will be based upon what is necessary for the medical care for the participant).

Referral to an appropriate health facility with the recommendation for transfusion if the participant is symptomatic with anemia. If the participant is not symptomatic, then clinical judgment may guide the recommendation for referral.

> -- Clinicians are advised to follow clinical guidelines for management of anemia in Botswana, and to base referral decisions for asymptomatic participants on these guidelines.

Participants should be monitored closely, with frequent (weekly until Grade 3, every 1-2 weeks thereafter) repeat haemoglobin testing (or a post-transfusion value to document resolution if transfusion is performed) until an anemia reaches Grade 2 or less.

Participants should be monitored clinically (by phone or visit) for signs/symptoms of infection until Grade 4 neutropenia reaches Grade 2 or less.

Once the anemia/neutropenia reaches Grade 2 or lower, study drug can be restarted with careful follow-up. If confirmed Grade 4 anemia/neutropenia recurs, then study CTX/placebo should be immediately and permanently discontinued, and not restarted.

8.3.4 Hepatic toxicities

Any infant in whom ongoing clinical hepatitis is diagnosed should have study CTX/placebo and NVP held (and alternative causes of hepatitis should be investigated, as indicated). Clinical hepatitis is generally defined as clinical signs and symptoms of clinical hepatic dysfunction regardless of ALT/AST values, including enlarged and tender liver, jaundice, and/or ascites.

If an etiology for the clinical hepatitis other than study drug is likely and ALT/AST is grade 1 or lower, then study CTX/placebo and NVP can be reintroduced sequentially with careful follow-up, in consultation with the study team.

8.3.5 Guidelines for Other Toxicities (other than rash, anemia/neutropenia, and hepatitis)

In general, study drugs may be continued for other toxicities that are Grade 1 or 2.

For Grade 3 toxicities, study drugs may be continued or held, depending on the toxicity and potential relatedness to study drug.

Participants who develop a symptomatic Grade 4 adverse event or toxicity felt to be related or possibly related to the study drug, and without alternative explanation, will have all study treatment withheld until resolution of the adverse event to a Grade ≤ 2.

Participants experiencing adverse events requiring permanent discontinuation of study treatment should be followed weekly until resolution of the adverse event to Grade ≤ 1 or until stabilized and no longer in need of such frequent monitoring, as determined by the site investigator.

Participants with Grade 4 asymptomatic laboratory abnormalities (other than anemia or neutropenia or ALT/AST) may continue study treatment after discussion with the protocol team only if the site investigator has compelling evidence that the toxicity is NOT related to the study treatment.

In cases in which study treatment was stopped due to a toxicity, it may be resumed (with careful monitoring) when that toxicity has resolved to Grade 2 or lower (other than in the instances of rash, hematologic abnormality or hepatitis described above).

8.4 Criteria for Study Treatment Discontinuation

- Drug-related toxicity
- Failure by the participant to attend 3 consecutive clinic visits.
- Participant repeatedly noncompliant with study treatment as prescribed, as determined by the site investigator.
- Clinical reasons believed life threatening by the site investigator, even if not addressed in the toxicity management of the protocol.
- Completion of study treatment as defined by protocol.

8.5 Criteria for Study Discontinuation

- Request by the participant to withdraw.
- Request of the primary care provider if s/he thinks the study is no longer in the best interest of the participant.

- Participant judged by the investigator to be at significant risk of failing to comply with the provisions of the protocol as to cause harm to self or seriously interfere with the validity of the study results.
- At the discretion of the NIH, local ministry of health, investigator, or pharmaceutical supporter.
- The study may be discontinued at any time by NIH, the IRB/ECs, Office of Human Research Protection (OHRP), Botswana Government or other government agencies as part of their duties to ensure that research participants are protected.

9.0 STATISTICAL CONSIDERATIONS

9.1. Primary Endpoints and Primary Analysis

The first primary endpoint is survival between randomization and 18 months for the comparison of CTX to placebo. A modified intent-to-treat approach will be taken: for the primary comparison of CTX to placebo, infants who have a positive HIV DNA PCR at the 4 week evaluation will be excluded (note that infants with a prior positive HIV test result will be excluded from the randomization). This exclusion is valid as the samples for testing are obtained prior to the intervention period and will be tested blinded to the randomized intervention assigned. Few HIV infections are anticipated to occur from 4 weeks to 18 months (~1%), but these few infants will be included in the endpoint. A secondary analysis will be performed that excludes these HIV-infected infants.

This primary analysis will be based on the Kaplan-Meier estimate of survival at 18 months of age, with time measured from the date of randomization, censoring subjects who are lost to follow-up at their last known date alive. All efforts will be made to establish vital status at 18 months of age. Sensitivity analyses will be conducted to assess the potential impact on the interpretation of results if children whose vital status at 18 months of age can not be obtained are considered to have died rather than have their follow-up censored.

9.2. Sample Size

The proposed sample size of 3,724 infants was chosen to give adequate power for the CTX vs. placebo randomized comparison. Based on data primarily from the Mashi study, but supported by preliminary data from the Mma Bana study, we estimate that the mortality rate between 4 weeks and 18 months among children who would be eligible for the randomized trial will be ~5.2% (for all feeding strategies combined) if assigned to receive the placebo. We believe that a 40% reduction in mortality from CTX is possible, and that this level of reduction would be of clinical importance. If the mortality rate between randomization and 18 months in the placebo arm is 5.2%, and if CTX reduces this rate by 40% irrespective of feeding choice, then the expected mortality in the CTX arm between randomization and 12 months will be 3.1%. Using a two-sided significance level of 0.05 in a continuity-corrected chi-square test, a sample size of 3,016 children will provide 80% power to detect a difference in mortality from 5.2% to 3.1%. This sample size is increased to 3,724 to allow for exclusions due to infant death and loss-to-follow-up before randomization (anticipated to be about 12%), HIV positivity by randomization (anticipated to be about 2%), and loss to follow-up after enrollment (about 5% based on our experience in other studies). Thus, we have conservatively allowed for ~ 19% losses in total, including pre-randomization and post-randomization losses.

We believe that randomizing infants to either CTX or placebo regardless of maternal choice of feeding strategy (FF or BF) or duration of BF (6 months vs. 12 months) is scientifically appropriate and ethical, and also generalizable for a mixed-feeding population in the real world. We have no reason to believe that the effect of CTX will differ whether infants are FF from birth, weaned at 6 months, or weaned at 12 months. All available evidence points to similar mortality rates and etiologies of mortality during early FF or after weaning from BF through 6 months, with the unifying explanation of risk being associated with the first period of life in which an infant is not breastfed (see Section B2). We hypothesize that the magnitude of mortality may be smaller with later weaning at 12 months, but the direction of the CTX effect would be the same. In the current Mma Bana Study, mortality rates are also similar across CD4 strata, and the use of maternal HAART does not appear to significantly modify infant mortality risk in comparison with the Mashi data (although this remains unanswered). Differences in the *causes* of mortality are not expected during the period from randomiztion to 18 months of life. Therefore, the potential for CTX to reduce mortality should be similar for each of the feeding groups. Furthermore, due to frequent infant HIV DNA PCR testing and very low rates of expected postnatal MTCT, we do not anticipate significant delays in initiating active CTX in children who become HIV-infected after randomization.

Sample size calculations for the randomized BF intervention suggest that there may be up to 80% power to detect a decrease in HIV infection or death between randomized arms from 6% to 3.5%, or a difference of ~42%, depending upon the proportion of women who choose to BF. These estimates assume the following event rates, for a total population of BF infants in which 50% receive CTX and 50% receive placebo: an 18-month mortality rate of 5% for 6 months of BF vs. an 18-month mortality rate of 2% for 12 months of BF; an 18-month HIV infection rate of 1% for 6 months of BF vs. an 18-month HIV infection rate of 1.5% for 12 months of BF. These mortality assumptions are partially supported by the Mma Bana Study, where most of the mortality that occurred beyond the first month of life was after weaning in the first year, and we hypothesize that this will be mitigated substantially among infants who do not wean until 12 months. For HIV-infection rates, the assumptions are based on data from the Mma Bana Study and recent infant NVP trials, considering only the rates while infants received NVP prophylaxis beyond 1 month and up to 6 months; from 6-12 months, a lower rate is assumed because infants likely to fail prophylaxis may do so within 6 months, but this is highly speculative and no data exist to guide this estimate. Given these assumptions, there will be 80% power to detect this true difference with an estimated total sample size of 2,428 BF infants with complete follow-up. This number would allow for 19% of infants in the study to FF and an additional 8% loss to follow-up for this analysis.

The 42% difference described above is for an intent-to-treat endpoint, which includes all infants who were randomized to this aspect of the study regardless of how feeding actually occurred. For the intent-to-treat analysis, the magnitude of the effect of the intervention may appear to be lower if there is a large amount of non-compliance. For the secondary “as treated” analysis, power to detect a true difference will be reduced if there is substantial non-compliance. For example, if only 1,746 infants succeed in feeding as assigned (30% loss, in addition to other calculated losses for death, HIV, and choice of formula feeding from birth), then there will be ~ 65% power to detect a true difference from 6% to 3.5%. However, as noted below, the 95% confidence intervals for differences of public health significance would remain relatively narrow.

9.3. Study Interpretation

9.3.1 CTX Randomization.

We believe that powering the study for a 40% difference represents an appropriate balance between study feasibility and public health significance, especially given the hypothesized mortality rates for this study. A 40% relative reduction in mortality from CTX, and an absolute reduction in mortality of 2.1% (from 5.2% to 3.1%), is biologically feasible. It is less than the mortality effect observed in several trials among HIV-infected individuals, and it would be a clinically meaningful finding. Even if a true difference in mortality exists but is less than the 2.1% absolute difference that this study is well-powered to detect, this difference may have less public health relevance if outweighed by considerations of cost, feasibility, competing interventions, or concerns about resistance and toxicity. If mortality rates during this intervention period are higher than 5.2% and 3.1% (as in many other African studies), the power to detect a true relative reduction of 40% will increase. Of note, although different in population and intervention design, the proposed NIH-funded PROMISE Study hypothesizes a 50% mortality reduction for CTX used after *later* weaning of 6% to 3%. If a 50% mortality reduction is accurate (as hypothesized in PROMISE), then our sample size will provide more than 95% power to detect a reduction from 5.2% to 2.6%.

We believe that a strength of this study is its ability to detect a small *absolute* difference in mortality at almost any realistic mortality rate that might be observed, and that this will be useful from a public health perspective. If mortality rates are *higher* than anticipated in both arms, the power for this study to detect a smaller relative mortality difference between arms will increase. This ensures that we will retain the power to detect a true *absolute* mortality difference between arms of < 2.8% even if mortality rates are as high as 8% in the placebo arm. We believe that a mortality rate below 5.2% in the placebo arm is unlikely based on our experience in the Mashi and Mma Bana studies. If, however, this is truly the case, then we retain high power to detect a true absolute difference of ~2%. For example, if mortality in the placebo arm is only 4%, then we will have over 87% power to detect this difference. Smaller absolute differences are not likely to have public health relevance.

9.3.2 Feeding Randomization

More uncertainty surrounds the estimates used for the feeding randomization, but this randomization is likely to yield important information even if differences are smaller (or larger) than hypothesized. Mortality and MTCT data for different durations of BF in the setting of maternal or infant ARV prophylaxis are lacking, and all mortality estimates vary by locality. No study of BF for longer than 6 months has been performed in Botswana. Therefore, the assumptions made for this aspect of the trial are speculative. In addition, the success of this intervention depends upon our ability to get most women to adhere to their assigned feeding duration, and smaller differences between groups are expected if large numbers of women in both groups wean before 6 months, or if women in the 12 month arm wean between 6 and 12 months. *However, because of the large sample size of this trial, the feeding randomization will yield important information for almost any hypothesized mortality and MTCT estimate.* If

differences between feeding arms are lower than estimated, the study is well-powered to support the alternate hypothesis that there is no benefit to extending the recommended BF period from 6 to 12 months. For example, if the overall HIV-free survival is 5% and equal in both arms, we would have the ability to exclude a true difference in rates between groups of +/- 1.7% with 95% confidence. With 30% losses for non-compliance, if HIV-free survival is 5% and equal in both arms, we would have the ability to exclude a true difference in rates between groups of +/- 2.1% with 95% confidence. Differences larger than this would have little public health significance.

9.4 Secondary Analyses

9.4.1. Randomized Duration of Breastfeeding.

An important secondary endpoint is HIV-free survival between randomization and 18 months among infants randomized to either 6 months or 12 months of BF. A modified intent-to-treat approach will be taken: for the primary comparison of HIV-free survival, infants who have a positive HIV DNA PCR at the 2-4 week evaluation will be excluded (note that infants with a prior positive HIV test result will be excluded from the randomization). This exclusion is valid as the samples for testing are obtained prior to the intervention period and will be tested blinded to the randomized intervention assigned.

This analysis will be based on the Kaplan-Meier estimate of survival at 18 months of age, with time measured from the date of randomization, censoring subjects who are lost to follow-up at their last known date alive. All efforts will be made to establish vital status and HIV status at 18 months of age. Sensitivity analyses will be conducted to assess the potential impact on the interpretation of results if children whose vital status or HIV status at 18 months of age cannot be obtained are considered to have died or become HIV-infected rather than have their follow-up censored.

This analysis will evaluate the overall strategy of promoting 6 vs. 12 months of total BF. Because it is expected that up to 30% of infants may wean prior to their intended date of weaning based on their randomized assignment (women may not be able to sustain BF for the intended duration, or may be counseled to wean early if HIV prophylaxis to mother or infant is discontinued), secondary “as treated” analyses will also be performed and will be presented as a corollary to the modified intent-to-treat analysis.

9.4.2. CTX Randomization Stratified by Feeding Method.

An important secondary objective is to compare rates of survival (and HIV-free survival) from *birth* to 18 months between the CTX and placebo arms, by chosen feeding method (any BF vs. FF) and by duration of BF (6 months vs. 12 months), although we do not anticipate that feeding will ultimately affect the direction of any benefit of CTX. Although the study is not specifically powered to compare CTX to placebo within subgroups defined by feeding, the study will provide reasonable precision to help guide public health policy decisions about the relative contributions of the feeding strategies and use of CTX on survival (and HIV-free survival). The addition of the HIV-negative women and their infants will provide important background mortality rates to help interpret these analyses.

9.4.3. Evaluation of the Breastfeeding MTCT Rate in the Setting of Extensive Prophylaxis

Given the large numbers of women expected to both BF (with prophylaxis) and FF, we can observationally confirm the low BF MTCT rate identified in the Mashi Study between birth and 4 weeks of age in the setting of BF + maternal antepartum/peripartum prophylaxis and infant prophylaxis through 4 weeks. In Mashi, we identified similar low MTCT rates among BF and FF infants through 4 weeks of age (1.3% and 1.1%, respectively), within a 95% confidence interval for the difference in rates of +/- 1.3%. Although this same comparison will not be randomized in this study, we will have excellent power to confirm the Mashi findings. More importantly, we will have a cohort of infants receiving MTCT prophylaxis through 6 or 12 months while BF, and with frequent PCR testing we can observationally evaluate the efficacy of these strategies over time.

9.4.4. Feeding objectives

Important secondary analyses for the feeding aspect of this study include and "as treated" analysis of survival and HIV-free survival by BF duration. We will also compare growth parameters and major morbidity by feeding arm and by the actual duration of BF (as treated). Because MTCT prophylaxis interventions have not been studied beyond ~ 6 months, it will also be important to document MTCT rates and potential toxicities (by prophylaxis strategy) at all time points, particularly those between 6-12 months.

9.4.5. AFASS Assessment

An exploratory analysis is planned to determine if the AFASS assessment used at study enrollment (which will be based on that of the Good Start Study and on updated WHO guidelines) predicts survival and HIV-free survival among those who initially decide to either FF or BF.

10.0 Human Subjects Considerations

10.1. Risks to the Subjects

10.1.1. Human Subjects Involvement and Characteristics

Human subjects will be enrolled in this study. HIV-infected pregnant women and the infants born to them will be enrolled. Involuntarily incarcerated individuals will not be enrolled. The health status of the HIV-uninfected children is expected to be similar to that of the general population. The health status of the HIV-infected children and of the HIV-infected mothers will reflect that of the large population of HIV-infected persons in Botswana. HIV-infected women or infants who meet treatment requirements will be referred to the Botswana Masa Programme for antiretroviral treatment (HAART).

Inclusion criteria for this study are based upon Botswana PMTCT and treatment policy, and in all cases the best achievable standard of care for women in Botswana will be employed. Non-Botswana citizens will not be enrolled, until such time as subsequent free treatment for medical conditions (such as HIV-1, depression etc) through the public sector can be guaranteed for non-citizens. The primary objectives of this study relate to pediatric outcomes, therefore children and their mothers are the target population. All enrollment will occur in Botswana, at established BHP clinical research sites.

10.1.2. Sources of Materials

Sources of data will include existing data from medical records; current participant recall/interview; and laboratory testing per protocol (all described in study procedures, above).

Data and laboratory samples will be linked to each participant by a numeric, unique participant identification number, which will not include any unique identifiers. The participant name/address/telephone number will protected by strict confidentiality procedures.. Items such as date of birth and study site will be included in the database. Only study staff that are immediately involved in the care/enrollment of the study participants will have access to unique subject identifiers.

10.1.3. Potential Risks

The IRBs that review this protocol will assess its risk categorization. It is our judgment that this protocol belongs in Category One Research under 45 CFR § 46 Subpart D: research involving greater than minimal risk but presenting the prospect of direct benefit to the individual subjects, based on the definitions of minimal risk in 45 CFR § 46.405. We believe that our IRBs will support our belief that: (a) the risk is justified by the anticipated benefit to the subjects; (b) the relation of the anticipated benefit to the risk is at least as favorable to the subjects as that presented by available alternative approaches; and (c) adequate provisions will be made for soliciting the assent of the children and permission of their parents or guardians, as set forth in §46.408.

Potential risks include psychological stress that might arise from a new diagnosis of HIV-1 (although most of the mothers will already know their HIV status), or from stigma associated with **participation at sites potentially associated with HIV care**. There is risk of unsafe FF use **(though FF will be by maternal choice)**, toxicity from either NVP or CTX. There is a risk of PCP in HIV-infected and not receiving CTX. There is a minor risk of local bruising and bleeding with phlebotomy. This study is not likely to pose any physical, social, or legal harm to participants.

10.2. Adequacy of protection against risks

10.2.1. Recruitment and Informed Consent

Mothers will undergo an informed consent process and sign a written informed consent for their and their child's participation in this study. The study informed consent form will contain all of the required elements as outlined by CFR 45, 46.116. Informed consent forms for BHP studies are translated into Setswana, and back-translated into English. These translations are approved by both IRBs. In general, Setswana-speaking nurses conduct the informed consent process with potential participants. In a private setting, the nurse verbally reviews the contents of the entire informed consent form with the participant (regardless of her literacy level). For more complicated studies, this process can take 1-2 hours, allowing sufficient time for participants to ask questions. Schematics/diagrams are used when possible. Sometimes, informed consent occurs over two separate visits. At the end of the discussion regarding the study (and full review of the informed consent form), the participant is given the opportunity to read the form, to ask questions, etc. Then, the nurse and study physician usually review the volunteer's understanding of study purpose, procedures, risks, benefits, etc.—the most important elements of the study from a human subjects perspective—to ensure that the volunteer understands these prior to

signing of the consent form by the participant and the study staff member who conducted the informed consent process.

10.2.2. Protection Against Risk

Confidentiality: All subjects enrolled in the study will be assigned a unique participant ID number. The participant ID number will be used for identification purposes on all laboratory specimens, evaluation forms, and reports retained in the research records. A list linking the subject names with the participant ID number will be stored at the clinical site under double locks, separate from all other research records. The only individuals who will have access to these lists will be study staff directly working on this study, and monitors/IRBs as needed. All research records will be stored in a secured area in locked files.

All research staff persons at the clinical sites are required to sign confidentiality forms pledging to hold research information in confidence. Furthermore, all study staff who work with human subjects undergo training in maintaining confidentiality (with SOPs specific to this topic).

Stigma: The risk of stigma is considered to be minor from participation in this study, and is mainly associated with willingness to be seen at BHP study clinics. This is minimized by the fact that this study will also enroll and provide care to non-HIV infected women and their infants at these sites. In previous studies, women who have joined have not later had concerns about being seen at BHP sites. Stigma may also occur from formula feeding to avoid HIV transmission, but this study will be *less* likely to cause such stigma than the government's national PMTCT program which recommends formula feeding from birth. In this study, only a portion of women would FF from birth, and this is by maternal choice. BF women would be indistinguishable in the maternity ward and in the community from HIV-negative women.

Safety of Infant Formula: FF is the standard of care in Botswana for HIV-exposed infants. There is a general belief that safe water sources and fuel availability exist for the safe preparation of formula. Although the Mashi Study led to concerns about FF safety at the 7 month endpoint, the mortality difference between FF and BF did not remain significant by 12 months. It is unknown whether early FF risk is from a modifiable ability to improve formula preparation vs. the *lack* of BF (antibodies and other protective factors) when FF occurs. We will take all steps possible to ensure that the former is not the case by creating a standardized questionnaire to assess AFASS for each potential participant. There are limited data to guide such a questionnaire, but the Good Start Study from South Africa suggests that access to safe water, available fuel, and HIV disclosure can predict safe use of infant formula [43]. We expect few women at our study sites will fall into a clearly non-AFASS category. For women who meet most basic AFASS requirements and who choose to FF, this choice will also be supported with education regarding safe formula feeding at each visit. These include direct counseling by nurses and research assistants, including the use of diagrams and standardized educational tools to promote correct preparation and administration.

Drug Toxicity: NVP and CTX will be considered study drugs. Both have well-described toxicity profiles. These study drugs have been used previously in the same clinical settings.

NVP has been used for 6-14 weeks of ongoing infant prophylaxis in 2 recent international clinical trials without significant toxicity. In the PEPI-Malawi Study, SAEs we not significantly

increased between the arms that received extended NVP vs. single-dose NVP at birth (N=609) [29]. In the SWEN Study, there was also no difference in SAEs for these comparator groups (N=1887) [32]. These comparisons included rash, neutropenias, and elevated LFTs. Based on these findings, we believe there is minimal risk of toxicity from the use of extended single-dose NVP in the first 12 months of. In contrast, extended ZDV for 1 month, which is the current standard in Botswana, may be associated with risk for transient infant neutropenias and anemias. Thus, we expect to see fewer infant neutropenias and anemias in this study during the first month of life when compared with previous studies and program data from Botswana where the standard-of-care was used.

CTX is the most common antibiotic used in African infants, and it may be associated with a small risk of neutropenia and anemia. CTX is recommended by the World Health Organization for use as prophylaxis against PCP among all HIV-exposed infants who are breastfeeding or who do not have access to PCR testing to confirm their HIV status, so it is used widely throughout Africa as prophylaxis in infants. In order to assess the safety of CTX use in the study, full blood counts (including differentials) will be drawn regularly, and also among all ill infants or infants suspected to be anemic. A detailed SOP exists for safely managing clinically important neutropenias and anemias.

Whether it is safe NOT to use CTX for all infants in the study also warrants discussion. We hypothesize that CTX will benefit HIV-exposed but uninfected infants, but this has not been proven in any setting to date. Thus, given that CTX has only been proven to be of benefit for HIV-*infected* infants, we believe it is appropriate and safe to use placebo for HIV-uninfected infants.

In Version 2.0 of this protocol, the allowable age for randomization to CTX/placebo has been extended from 28-34 days to 14-34 days for full-term infants. This decision has been made after careful consideration of the risks and benefits. The benefit is the extension of a potentially beneficial intervention (CTX) to infants who are experiencing the highest risk of mortality during the first month of life. This has the potential to improve infant survival, particularly among FF infants, who are at the greatest risk during the first month of life. In Version 1.0 of this protocol, there was an expectation that most infants would be BF and have protection early in life by maternal antibodies and the known benefits of early breastfeeding. However, we now expect a higher proportion of FF infants in the study, and this shifts the greatest risk period form after weaning (as anticipated in BF infants) to the first month of life (the first period without maternal protection). In the Mashi Study, 3.4% of all FF infants died during the first month of life, and approximately 1% after the second week. Thus, starting CTX earlier has the potential for significant mortality benefit, and it will improve the power of the study overall if the estimated mortality rates between 4 weeks and 15 months are lower than originally anticipated.

There are 3 safety considerations in starting CTX earlier, and all have been addressed in the current version of the protocol.

The first consideration is for kernicterus risk among preterm infants. Sulfa drugs may have a risk for contributing to kernicterus when used in *preterm* infants during the neonatal period, and there is a theoretical risk for preterm infants extending throughout the first month of life. The only data for this is from a 1956 study by Silverman et. al. [45] that demonstrated increased risk of

kernicterus and death among infants admitted to the prematurity ward (median birthweights of 1.5 kg) who received sulfisoxazole within 120 hours of birth. The dose of sulfisoxazole in this study was dosed higher than current recommendations. In contrast, no studies performed among term infants have suggested excess kernicterus with sulfa agents. Treatment with pyrimethamine and sulfadiazine for 1 year beginning at birth became the standard of care for congenital toxoplasmosis in the 1980s, and no case series have reported no cases of kernicterus in >800 newborns treated [46]. WHO recommends the use of CTX as the first-line treatment for respiratory illness in the developing world among non-jaundiced, non-premature neonates [47], and several large trials in India have reported no excess jaundice/kernicterus with CTX use in neonates [48]. Finally, CTX has been used extensively during the third trimester of pregnancy (and crosses the placenta well) without reported kernicterus among newborns [49].Given the above considerations, we believe there may be a very small risk for kernicterus early in life among preterm infants, and to address this, CTX will not be used until 2 weeks of life in any infant, and will not be used until 4 weeks of life for those born < 36 weeks gestation or in infants < 2.5 kg at 2 weeks.

The second consideration with earlier use of CTX is the possibility of overlapping bone marrow toxicity when used concurrently with ZDV. To avoid this problem, we will now use NVP for all infants in the study, rather than only the BF infants. NVP is the preferred agent for infant HIV prophylaxis by WHO[50], does not lead to excess anemia when used for PMTCT [51-52], and adds no apparent excess risk for hematologic toxicity when used with CTX.[53].

The last consideration with starting CTX at 2 weeks is the dosing. PK studies demonstrate low sulphamethoxazole clearance in neonates [54], but there is no evidence that this occurs by 2 weeks of age. WHO and other guidelines recommend 1.25 ml *twice* daily of 200mg/40mg per 5 ml suspension for infants < 1 month of age, and 2.5 ml *twice* daily at 1 month [47-48]. Version 1.0 of the protocol has used 2.5 ml once daily starting at 1 month. Because the difference in weight between 2 weeks and 1 month is expected to differ by < 30%, and because dosing in this study is half of the recommended treatment dose, we have chosen to use 2.5 ml once daily starting at 2 weeks for infants born >/= 36 weeks gestation (therefore 38 weeks equivalent by 2 weeks of life) and > 2.5 kg. Infants born < 36 weeks gestation or who are <2.5 kg at 2 weeks of age will not begin study drug until >/= 28 days of life, as is the current WHO-recommended practice.

<u>MTCT from BF</u>: HIV-infected women who choose to BF will be at some risk for transmitting HIV to their infants. In the Mashi Study, we found no appreciable risk of BF MTCT in the first month of life (identical birth to 4 week positivity between FF and BF arms). Beyond 4 weeks, late MTCT occurred in Mashi in the setting of ZDV prophylaxis – 2.7% from 4 weeks to 4 months, and 1.7% from 4 to 7 months. However, NVP prophylaxis may be superior to ZDV prophylaxis. In the PEPI-Malawi Study, the additional risk of MTCT between 6 weeks and 14 weeks was only 1.1%. In the BAN Study, similar low MTCT risk was noted through 6 months.

The late MTCT risk from BF + NVP prophylaxis is likely balanced by the mortality benefit provided to the infant from BF. At the very least, the Mashi Study demonstrated similar 18-month HIV-free survival between feeding arms (with a potentially inferior prophylaxis agent than NVP). All HIV-infected women will be appropriately counseled about the risks/benefits of BF vs. FF to help each participant decide what the preferred method is for her individual

circumstances. If BF is chosen, we believe there is equipoise for randomizing to 6 vs. 12 months in the setting of prophylaxis. Formula (in the first year) and safe weaning foods will be made available whenever women in the study decide to wean.

Drug Resistance: There are resistance considerations for both NVP and CTX. For the use of extended NVP, there is a small but real risk that any infants who become infected from early breastfeeding would be more likely to develop a NVP-resistant virus [29]. This could limit treatment options, although treatment could still be successfully implemented with non-NNRTI regimens. However, we expect breastfeeding transmission to be extremely rare in this study – extended NVP should be as effective or more effective than ZDV, and in the Mashi Study we detected little or no BF transmission during the first month of life (see Section B above). For those women who BF while giving infant NVP, there may be an additional MTCT risk based on the PEPI-Malawi Study findings [29]. NVP resistance would be expected in 40-87% of those receiving the standard-of-care single-dose NVP, which persists during most of the first month of life in infant blood [30] [55-56]. Thus, we believe that if resistance is increased because of early use of extended NVP prophylaxis compared with single-dose NVP, the absolute difference would be small, it would affect fewer than 1% of infants, and it would be offset by the lower PMTCT and lower toxicity benefit of using NVP rather than ZDV for this extended prophylaxis (and potentially lower mortality than with the use of FF from birth).

CTX resistance is an ongoing concern in Africa because of its wide use among infants, children, and adults [57]. The consideration of the recommendation by WHO for the use of CTX as prophylaxis among HIV-exposed infants has taken resistance into account and most experts believe that the *benefit outweighs the risk* of its use [58]. We agree with this assessment. For major illnesses (excluding PCP), CTX cannot be considered to be reliably active against all pathogens, and therefore it is reasonable to use it primarily as prophylaxis (where even partial activity may help defend against a pathogen that is unestablished). High rates of resistance are reported for diarrheal and respiratory pathogens throughout Africa [59]. In the treatment setting, clinicians should consider alternative options, as we will do for ill infants in this study. Data support this approach; in the HIV-infected setting prophylactic CTX has shown clear benefits for reducing the risks of pneumonias and diarrhea and mortality from non-PCP causes [15, 24]. This has been found even where resistance to CTX is relatively common [16]. This is an important consideration in our choice of CTX as a prophylactic agent.

We hypothesize that the same benefits will be demonstrated among HIV-exposed but uninfected infants, but the benefit of prophylaxis vs. the risk from resistance and toxicity (and the cost of prophylaxis) will be evaluated by the study. As a secondary objective, we will perform resistance testing among clinically relevant pathogens to explore the potential association with CTX vs. placebo.

CTX vs. Placebo During BF: A potential risk is that infants who become HIV-infected during breastfeeding may be randomized to the placebo arm and not receive CTX. However, we believe that this risk is negligible for the following reasons: First, data from several international trials (including preliminary data from our Mma Bana Study) [29, 32-34] suggest that the risk of postnatal transmission in this setting of maternal HAART or infant NVP prophylaxis will be < 1-2%. If infant NVP or maternal HAART are not being used reliably (or if plasma HIV-1 RNA is > 400 copies at 1 month for women receiving HAART) women will be counseled not to BF.

Second, we will check infant DNA PCR for HIV at frequent intervals, and any infected infants will immediately receive CTX prophylaxis. Infants infected via BF would not be expected to develop PCP within such a short time interval. Any infant with symptoms concerning for HIV infection between study visits will receive more frequent HIV PCR testing. Finally, for the infants whose mothers are on HAART, the potential for additive hematologic toxicities among BF infants receiving CTX whose mothers are also receiving HAART could outweigh any potential benefits of CTX use. Although we do not anticipate significant additive toxicities (low total doses of NRTIs are expected from breastfeeding [60]), the randomized design will allow for precise toxicity evaluation and close DSMB monitoring of risk, and will allow us to evaluate this important safety question. Thus, we believe that if there is any risk for PCP at all, there is equipoise between this very small potential risk of PCP *without* CTX vs. the very small risk of life-threatening anemias *with* CTX to warrant the randomization to either CTX or placebo for BF infants.

10.3. Potential benefits of the proposed research to the subjects and others

The intent of this study is to identify an improved strategy for overall survival and HIV-free survival of infants born to HIV-infected women. No infant will receive less than the current best standard of care in Botswana. HIV-exposed infants may benefit from protection against MTCT from NVP prophylaxis (with less toxicity than ZDV), and CTX may improve infant survival for those receiving the active drug. Placebo will have no effect on those infants who receive it. Frequent HIV testing will detect HIV-infected infants who require active CTX prophylaxis before PCP becomes a concern**.** Longer BF may improve overall infant HIV-free survival. All infants will likely benefit from improved overall medical care and access to nurses and physicians through this study.

10.4. Importance of the knowledge to be gained

This study has broad applicability for Botswana and for other regions of the world. The CTX intervention is applicable to all HIV-exposed infants and could improve survival in almost any setting. Although not directly studied, the results of this study could inform about the importance of using CTX at later ages as well. The 6 vs. 12 months BF randomization is important and novel, as there are no data to guide appropriate duration of BF in the setting of prophylaxis.

This clinical trial will inform public health policy in Botswana, Africa, and throughout the developing world.

10.5. Inclusion of women and minorities

Pregnant and postpartum women will comprise 100% of those women enrolled in the study. BHP study sites are accessible to individuals drawn from different ethnic groups within Botswana, all of whom have access to government clinics and who are offered HIV testing by the National Program to prevent MTCT. Our study enrollees therefore should include minority ethnic groups from within Botswana, but all would be classified as Black African.

10.6. Inclusion of Children

Children are included in this research plan, and will be followed and provided care until 18 months of age. Dr. McIntosh served as Chief of the Division of Infectious Diseases at Children's Hospital in Boston for 20 years. Drs. Shapiro and Lockman have spent 10 years working on projects in Botswana designed to prospectively follow infants born to HIV-infected mothers and to diagnose and treat their respiratory and diarrheal illnesses and bloodstream infections. Children who are HIV-positive will be treated with antiretrovirals through the Botswana Government. Routine care of children, including immunizations, will occur either at our Study Clinic or at local Government health clinics (if staffing constraints limit the amount of clinical care that can be provided as these research sites).

10.7. Monitoring and Interim Analyses

The study is being monitored regularly by an independent Data and Safety Monitoring Board (DSMB) comprised of individuals based both in the US and in Africa, specifically the standing NIH "Africa" DSMB (this is the DSMB that monitored the Mma Bana Study). Data such as enrollment, visit compliance, follow-up, laboratory evaluations, data submission, and quality control will be presented.

The DSMB will monitor the occurrence of any adverse effects. Safety and efficacy parameters will initially be reviewed approximately 6-8 months after the first infant is randomized, and then every 6-12 months unless otherwise recommended by the DSMB. If unexpected serious adverse events judged possibly related to treatment are reported at any time, the team will request additional review by the DSMB. Additional reviews or an altered schedule of review or trigger for early review may be instituted at the discretion of the DSMB. The DSMB will make recommendations at each of these reviews regarding whether the study should continue as originally designed. Statistical analyses will be prepared in advance of each DSMB meeting.

Peto-Haybittle stopping guidelines will be used in efficacy analysis for the randomized comparisons, requiring $p<0.001$ before stopping in favor of any randomized arm, because of the public health significance of the study and the fact that the study is unlikely to be replicated and needs to be definitive. Termination of the CTX arm or longer BF duration could be considered by the DSMB with less definitive evidence if mortality results suggest an adverse effect on survival or HIV infection. In the absence of safety issues, the randomized comparisons would not be terminated early if no differences are detected between arms as maximizing precision to establish such a finding would have public health importance.

10.8. Biohazard containment

As the transmission of HIV and other blood-borne pathogens can occur through contact with contaminated needles, blood, and blood products, appropriate blood and secretion precautions will be employed by all personnel in the drawing of blood and shipping and handling of all specimens for this study, as currently recommended by the Centers for Disease Control.

All infectious specimens will be sent using the ISS-1 SAF-T-PAK mandated by the International Air Transport Association Dangerous Goods Regulations Packing Instruction 602. Please refer to individual carrier guidelines (for example: Federal Express, Airborne, etc.) for specific instructions.

11.0 LITERATURE CITED

1. Thea DM, St. Louis ME, Atido U. A prospective study of diarrhea and HIV-1 infection among 429 Zairian infants. *N Engl J Med* 1993,**329**:1696-1702.
2. Brahmbhatt H, Kigozi G, Wabwire-Mangen F, Serwadda D, Lutalo T, Nalugoda F, *et al*. Mortality in HIV-infected and uninfected children of HIV-infected and uninfected mothers in rural Uganda. *J Acquir Immune Defic Syndr* 2006,**41**:504-508.
3. Marinda E, Humphrey JH, Iliff PJ, et al. Child mortality according to maternal and infant HIV status in Zimbabwe. Program and abstracts of the XVI International AIDS Conference; August 13-18, 2006; Toronto, Canada. Abstract THAC0203.
4. Newell ML, Coovadia H, Cortina-Borja M, Rollins N, Gaillard P, Dabis F. Mortality of infected and uninfected infants born to HIV-infected mothers in Africa: a pooled analysis. *Lancet* 2004,**364**:1236-1243.
5. Thior I, Lockman S, Smeaton LM, Shapiro RL, Wester C, Heymann SJ, *et al*. Breastfeeding plus infant zidovudine prophylaxis for 6 months vs formula feeding plus infant zidovudine for 1 month to reduce mother-to-child HIV transmission in Botswana: a randomized trial: the Mashi Study. *JAMA* 2006,**296**:794-805.
6. Creek T, Arvelo W, Kim A, Lu L, Bowen A, Finkbeiner T, Zaks L, Masunge J, Shaffer N, and Davis M. Role of Infant Feeding and HIV in a Severe Outbreak of Diarrhea and Malnutrition among Young Children, Botswana, 2006. 14th Conference on Retroviruses and Opportunistic Infections, Los Angeles, 25-28 Feb 2007 (Abstract 770).
7. Sinkala M, Kuhn L, Kankasa C, Kasonde P, Vwalika C, Mwiya M, Scott N, Semrau K, Aldrovandi G, Thea D, and Zambia Exclusive Breastfeeding Study Group (ZEBS). No Benefit of Early Cessation of Breastfeeding at 4 Months on HIV-free Survival of Infants Born to HIV-infected Mothers in Zambia: The Zambia Exclusive Breastfeeding Study. 14th Conference on Retroviruses and Opportunistic Infections, Los Angeles, February 25-28, 2007. Abstract 74.
8. Kafulafula G, Thigpen M, Hoover D, Li Q, N Kumwenda N, Mipando L, Taha T, Mofenson L, and Fowler M. Post-weaning Gastroenteritis and Mortality in HIV-uninfected African Infants Receiving Antiretroviral Prophylaxis to Prevent MTCT of HIV-1. 14th Conference on Retroviruses and Opportunistic Infections, Los Angeles, February 25-28, 2007. Abstract 773.
9. Lockman S, Smeaton L, Shapiro R, Thior I, Wester C, Stevens L, Moffat C, Ndase P, Arimi P, Asmelash A, Owor A, Makhema J, Essex M, Mashi Study Team. Morbidity and mortality among infants born to HIV-infected mothers and randomized to breastfeeding versus formula-feeding in Botswana (Mashi Study). XVI International AIDS Conference, Toronto Canada, 13-18 August 2006. Abstract # TUPE0357.
10. Lockman S, Smeaton L, Shapiro R, Thior I, Wester C, Makhema J, Ogwu A, Asmelash A, Widenfelt E, and Essex M. Risk Factors for and Timing of Infant Mortality among

HIV-exposed Children: A Randomized Clinical Trial in Botswana. 15th Conference on Retroviruses and Opportunistic Infections, Boston, February 3-6, 2008. Abstract 643.

11. Kuhn L, Aldrovandi GM, Sinkala M, Kankasa C, Semrau K, Mwiya M, *et al.* Effects of Early, Abrupt Weaning for HIV-free Survival of Children in Zambia. *N Engl J Med* 2008.
12. WHO. WHO Collaborative Study Team. Effect of breastfeeding on infant and child mmortality due to infectious diseases in less developed countries: a pooled analysis. *Lancet* 2000,**355**:451-455.
13. Wiktor SZ, Sassan-Morokro M, Grant AD, Abouya L, Karon JM, Maurice C, *et al.* Efficacy of trimethoprim-sulphamethoxazole prophylaxis to decrease morbidity and mortality in HIV-1-infected patients with tuberculosis in Abidjan, Cote d'Ivoire: a randomised controlled trial. *Lancet* 1999,**353**:1469-1475.
14. Anglaret X, Chene G, Attia A, Toure S, Lafont S, Combe P, *et al.* Early chemoprophylaxis with trimethoprim-sulphamethoxazole for HIV-1-infected adults in Abidjan, Cote d'Ivoire: a randomised trial. Cotrimo-CI Study Group. *Lancet* 1999,**353**:1463-1468.
15. Mermin J, Lule J, Ekwaru JP, Malamba S, Downing R, Ransom R, *et al.* Effect of co-trimoxazole prophylaxis on morbidity, mortality, CD4-cell count, and viral load in HIV infection in rural Uganda. *Lancet* 2004,**364**:1428-1434.
16. Chintu C, Bhat GJ, Walker AS, Mulenga V, Sinyinza F, Lishimpi K, *et al.* Co-trimoxazole as prophylaxis against opportunistic infections in HIV-infected Zambian children (CHAP): a double-blind randomised placebo-controlled trial. *Lancet* 2004,**364**:1865-1871.
17. Lowrance D, Makombe S, Harries A, Yu J, Aberle-Grasse J, Eiger O, Shiraishi R, Marston B, Ellerbrock T, and Libamba E. Cotrimoxazole Prophylaxis Reduced the Early Mortality of HIV-infected Patients on ART in Malawi. 14th CROI, 25-28 February 2007, Los Angeles. Abstract 83.
18. Theophil B, Kinabo G, Swai M, Shao J, Mchele J, Henderikxm M, Mulder B, Houpt E, Tolboom J, Schimana W. Positive Effect of Cotrimoxazole Prophylaxis in HIV-infected Children. 14th CROI, 25-28 February 2007, Los Angeles. Abstract 705.
19. Mbori-Ngacha D, Nduati R, John G, Reilly M, Richardson B, Mwatha A, *et al.* Morbidity and mortality in breastfed and formula-fed infants of HIV-1- infected women: A randomized clinical trial. *Jama* 2001,**286**:2413-2420.
20. Marinda E, Humphrey JH, Iliff PJ, Mutasa K, Nathoo KJ, Piwoz EG, *et al.* Child mortality according to maternal and infant HIV status in Zimbabwe. *Pediatr Infect Dis J* 2007,**26**:519-526.
21. McNally LM, Jeena PM, Gajee K, Thula SA, Sturm AW, Cassol S, *et al.* Effect of age, polymicrobial disease, and maternal HIV status on treatment response and cause of severe pneumonia in South African children: a prospective descriptive study. *Lancet* 2007,**369**:1440-1451.
22. Ryan M, Chitah B, Griffin S, Walker S, Mulenga V, Kalolo D, Thomason M, Chintu C, Sculpher M, and Gibb D. The Cost Effectiveness of Cotrimoxazole as Prophylaxis against OI in HIV-infected African Children: The CHAP Trial. 14th CROI, 25-28 February 2007, Los Angeles. Abstract 706.
23. Campbell J, Moore D, Degerman R, Kaharuza F, Were W, Muramuzi E, Mermin J, and Tappero1 J. HIV-infected Ugandans on HAART with CD4 Counts >200 Cells/mm3 Who

Discontinue Cotrimoxazole Have Increased Risk of Malaria and Diarrhea. 14th CROI, 25-28 February 2007, Los Angeles. Abstract 33.

24. Mermin J, Lule J, Ekwaru JP, Downing R, Hughes P, Bunnell R, *et al.* Cotrimoxazole prophylaxis by HIV-infected persons in Uganda reduces morbidity and mortality among HIV-uninfected family members. *Aids* 2005,**19**:1035-1042.
25. Coutsoudis A, Pillay K, Spooner E, Coovadia HM, Pembrey L, Newell ML. Routinely available cotrimoxazole prophylaxis and occurrence of respiratory and diarrhoeal morbidity in infants born to HIV-infected mothers in South Africa. *S Afr Med J* 2005,**95**:339-345.
26. Morris A, Lundgren JD, Masur H, Walzer PD, Hanson DL, Frederick T, *et al.* Current epidemiology of Pneumocystis pneumonia. *Emerg Infect Dis* 2004,**10**:1713-1720.
27. World Health Organization. Guidelines on co-trimoxazole prophylaxis for HIV-related infections among children, adolescents and adults: recommendations for a public health approach. WHO Press (2007), World Health
Organization, 20 Avenue Appia, 1211 Geneva 27, Switzerland. http://www.who.int/hiv/pub/guidelines/ctx/en/index.html.
28. Zachariah R, Harries AD, Luo C, Bachman G, Graham SM. Scaling-up co-trimoxazole prophylaxis in HIV-exposed and HIV-infected children in high HIV-prevalence countries. *Lancet Infect Dis* 2007,**7**:686-693.
29. Kumwenda NI, Hoover DR, Mofenson LM, Thigpen MC, Kafulafula G, Li Q, *et al.* Extended Antiretroviral Prophylaxis to Reduce Breast-Milk HIV-1 Transmission. *N Engl J Med* 2008.
30. Sastry J and The Six Week Extended Dose Nevirapine (SWEN) Study Team. Extended-dose Nevirapine to 6 Weeks of Age for Infants in Ethiopia, India, and Uganda: A Randomized Trial for Prevention of HIV Transmission through Breastfeeding.15th Conference on Retroviruses and Opportunistic Infections, Boston, February 3-6, 2008. Abstract 43.
31. Shapiro RL, Smeaton L, Lockman S, Thior I, Wester C, Rossenkhan R, Stevens L, Leidner J, Makhema J, Essex M. Risk Factors for Mother-to-Child Transmission of HIV-1 from Breastfeeding in a Randomized Clinical Trial in Botswana (the Mashi Study). *J Infect Dis* 2009;199:414-8.
32. Bedri A, Gudetta B, Isehak A, Kumbi S, Lulseged S, Mengistu Y, *et al.* Extended-dose nevirapine to 6 weeks of age for infants to prevent HIV transmission via breastfeeding in Ethiopia, India, and Uganda: an analysis of three randomised controlled trials. *Lancet* 2008,**372**:300-313.
33. Kilewo C, Karlsson K, Ngarina M, Massawe A, Lyamuya E, Lipyoga R, Msemo G, Swai A, Mhalu F, Biberfeld G. Prevention of mother to child transmission of HIV-1 through breastfeeding by treating mothers prophylactically with triple antiretroviral therapy in Dar es Salaam, Tanzania – the MITRA Plus study. Fourth International AIDS Society Conference on HIV Treatment and Pathogenesis, Sydney, July 22-25, 2007. Abstract TuAX101.
34. Arendt V, Ndimubanzi P, Vyankandondera J, Ndayisaba G, Muganda J, Courteille O, Rutanga C, Havuga E, Dhont N, Mujawamassiga A, Omes C, Peltier A. AMATA study: effectiveness of antiretroviral therapy in breastfeeding mothers to prevent post-natal vertical transmission in Rwanda. Fourth International AIDS Society Conference on HIV Treatment and Pathogenesis, Sydney, July 22-25, 2007. Abstract TuAX102.

35. Briend A, Wojtyniak B, Rowland M. Breast feeding, nutritional state, and child survival in rural Bangladesh. *Br Med J* 1988,**296**:879-882.
36. Howie PW, Forsyth JS, Ogston SA, Clark A, Florey CD. Protective effect of breast feeding against infection. *Br Med J* 1990,**300**:11-16.
37. Wright A, Holberg C, Martinez F, Morgan W, Taussig L. Breast feeding and lower respiratory tract illness in the first year of life. Group Health Medical Associates. *BMJ* 1989,**299**:946-949.
38. Becquet R, Bequet L, Ekouevi DK, Viho I, Sakarovitch C, Fassinou P, *et al.* Two-year morbidity-mortality and alternatives to prolonged breast-feeding among children born to HIV-infected mothers in Cote d'Ivoire. *PLoS Med* 2007,**4**:e17.
39. Omwega AM, Oguta TJ, Sehmi JK. Maternal knowledge on mother-to-child transmission of HIV and breastmilk alternatives for HIV positive mothers in Homa Bay District Hospital, Kenya. *East Afr Med J* 2006,**83**:610-618.
40. Otieno PA, Brown ER, Mbori-Ngacha DA, Nduati RW, Farquhar C, Obimbo EM, *et al.* HIV-1 disease progression in breast-feeding and formula-feeding mothers: a prospective 2-year comparison of T cell subsets, HIV-1 RNA levels, and mortality. *J Infect Dis* 2007,**195**:220-229.
41. South Africa Demographic and Health Survey 1998. http://www.measuredhs.com/pubs/pub_details.cfm?ID=360&srchTp=advanced.
42. HIV Transmission and Breast Feeding Task Group, Department of Health, Republic of South Africa. Policy guideline and recommendation for feeding of infants of HIV positive mothers. http://www.doh.gov.za/aids/docs/feeding.html.
43. Doherty T, Chopra M, Jackson D, Goga A, Colvin M, Persson LA. Effectiveness of the WHO/UNICEF guidelines on infant feeding for HIV-positive women: results from a prospective cohort study in South Africa. *Aids* 2007,**21**:1791-1797.
44. Chasela C, Hudgens M, Jamieson D, Kayira D, Hosseinipour M, Ahmed Y, Tegha G, Knight R, Kourtis AP, Kamwendo D, Hoffman I, Ellington H, Kacheche Z, Wiener J, Martinson F, Kazembe P, Mofolo I, Long D, Soko A, Smith SB, van der Horst C. Both maternal HAART and daily infant nevirapine (NVP) are effective in reducing HIV-1 transmission during breastfeeding in a randomized trial in Malawi: 28 week results of the Breastfeeding, Antiretroviral and Nutrition (BAN) Study. 5th International AIDS Society Conference on HIV Pathogenesis, Treatment, and Prevention, Cape Town, July 19-22, 2009. Abstract WELBC103.
45. Silverman WA, Andersen DH, Blanc WA, Crozier DN. Pediatrics. A difference in mortality rate and incidence of kernicterus among premature infants allotted to two prophylactic antibacterial regimens. Pediatrics 1956;4:614-25.
46. Peters PJ, Thigpen MC, Parise ME, Newman RD. Safety and toxicity of sulfadoxine/pyrimethamine: implications for malaria prevention in pregnancy using intermittent preventive treatment. *Drug Saf* 2007,**30**:481-501.
47. WHO. Management of Acute Respiratory Infections in Children. Geneva, 1995.
48. Bang AT, Bang RA, Baitule SB, Reddy MH, Deshmukh MD. Effect of home-based neonatal care and management of sepsis on neonatal mortality: field trial in rural India. *Lancet* 1999,**354**:1955-1961.
49. Forna F, McConnell M, Kitabire FN, Homsy J, Brooks JT, Mermin J, *et al.* Systematic review of the safety of trimethoprim-sulfamethoxazole for prophylaxis in HIV-infected pregnant women: implications for resource-limited settings. *AIDS Rev* 2006,**8**:24-36.

50. World Health Organization. Antiretroviral Drugs for Treating Pregnant Women and Preventing HIV Infection in Infants: Recommendations for a Public Health Approach – 2010 version. WHO Press, 2010. World Health Organization, 20 Avenue Appia, 1211 Geneva 27, Switzerland. .
51. Chasela CS, Hudgens MG, Jamieson DJ, Kayira D, Hosseinipour MC, Kourtis AP, *et al.* Maternal or infant antiretroviral drugs to reduce HIV-1 transmission. *N Engl J Med* 2010,**362**:2271-2281.
52. Taha T, Kumwenda J, Kafulafula G, Fowler MG, Chen S, Li Q, Hoover D, Thigpen M, Kumwenda N, Mofenson L. Maternal highly active antiretroviral treatment (HAART): does it improve child survival? Fifth International AIDS Society Conference on HIV Treatment, Pathogenesis and Prevention, Cape Town, July 19-22, 2009. Abstract MoPEB083.
53. Aizire J, Fowler MG, Wang J, Shetty AK, Stranix-Chibanda L, Kamateeka M, *et al.* Extended prophylaxis with nevirapine and cotrimoxazole among HIV-exposed uninfected infants is well tolerated. *AIDS* 2011.
54. Springer C, Eyal F, Michel J. Pharmacology of trimethoprim-sulfamethoxazole in newborn infants. *J Pediatr* 1982,**100**:647-650.
55. Martinson NA, Morris L, Gray G, Moodley D, Pillay V, Cohen S, *et al.* Selection and persistence of viral resistance in HIV-infected children after exposure to single-dose nevirapine. *J Acquir Immune Defic Syndr* 2007,**44**:148-153.
56. Eshleman SH, Hoover DR, Chen S, Hudelson SE, Guay LA, Mwatha A, *et al.* Resistance after single-dose nevirapine prophylaxis emerges in a high proportion of Malawian newborns. *Aids* 2005,**19**:2167-2169.
57. Hamel MJ, Greene C, Chiller T, Ouma P, Polyak C, Otieno K, *et al.* Does cotrimoxazole prophylaxis for the prevention of HIV-associated opportunistic infections select for resistant pathogens in Kenyan adults? *Am J Trop Med Hyg* 2008,**79**:320-330.
58. World Health Organization. Antiretroviral Drugs for Treating Pregnant Women and Preventing HIV Infection in Infants: Towards Universal Access: Recommendations for a Public Health Approach – 2006 version. WHO Press, 2006. World Health Organization, 20 Avenue Appia, 1211 Geneva 27, Switzerland. .
59. Shapiro RL, Kumar L, Phillips-Howard P, et al. Antimicrobial-resistant bacterial diarrhea in western Kenya. J Infect Dis 2001;183;1701-04.
60. Shapiro RL, Holland D, Capparelli E, et al. Antiretroviral Concentrations in Breastfeeding Infants of Women in Botswana Receiving Antiretriviral Treatment. J Infect Dis 2005;192:720-7.
