# Supplementary material for: Decreased diarrheal and respiratory disease in HIV exposed uninfected children following vaccination with rotavirus and pneumococcal conjugate vaccines
Source: PLoS One. 2020 Dec 21;15(12):e0244100. doi: 10.1371/journal.pone.0244100 (PMC7751865; doi:10.1371/journal.pone.0244100)
Supplement: Supplementary file 6 [file pone.0244100.s002.docx]

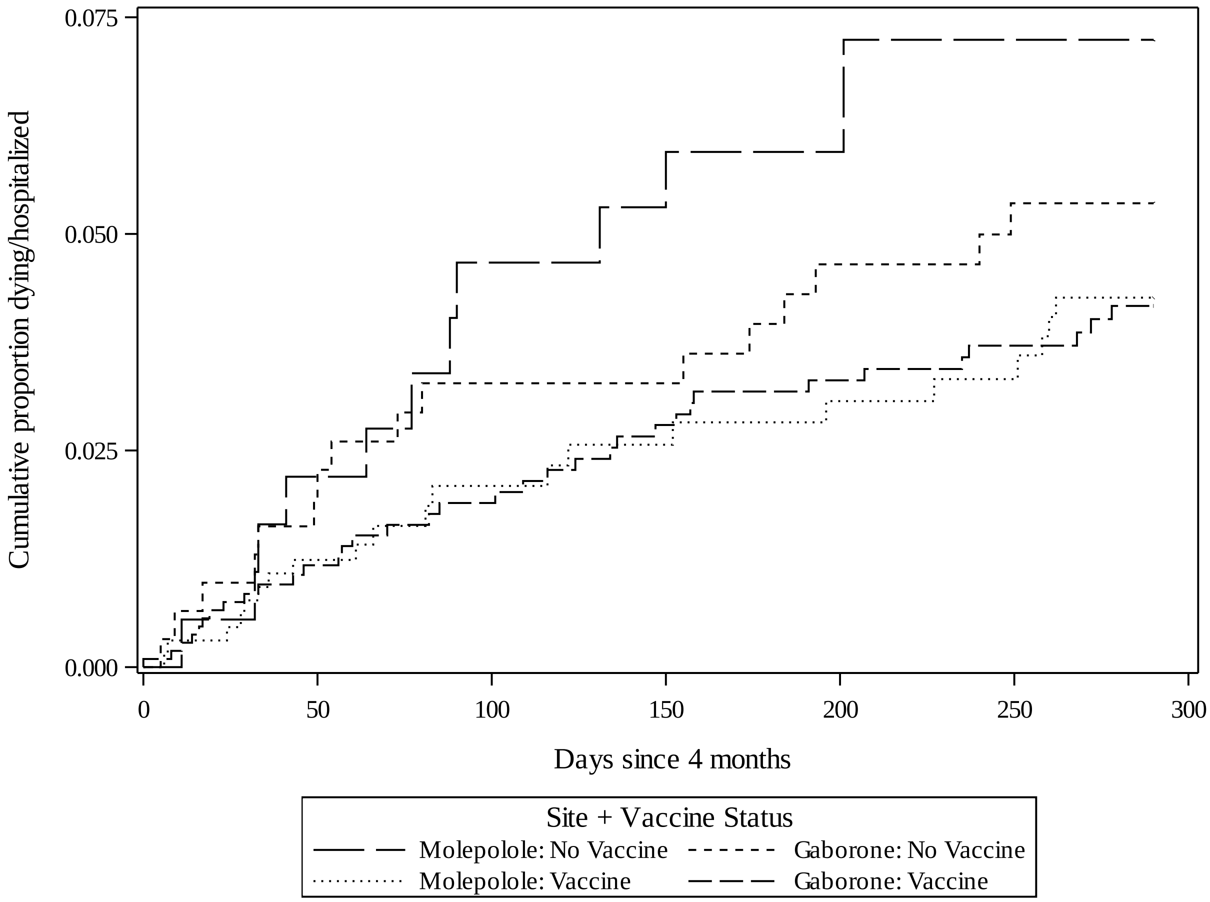

Cumulative proportion dying/hospitalized
0.000
0.025
0.050
0.075
0
50
100
150
200
250
300
Days since 4 months
Site + Vaccine Status
Molepolole: No Vaccine
Gaborone: No Vaccine
Molepolole: Vaccine
Gaborone: Vaccine
